# The effects of cannabinoid 1 receptor compounds on memory: a meta-analysis and systematic review across species

**DOI:** 10.1007/s00213-019-05283-3

**Published:** 2019-06-05

**Authors:** Faith Borgan, Katherine Beck, Emma Butler, Robert McCutcheon, Mattia Veronese, Anthony Vernon, Oliver D. Howes

**Affiliations:** 1 Department of Psychosis Studies, Institute of Psychiatry, Psychology and Neuroscience, King’s College London, 16 De Crespigny Park Road, London, SE5 8AF UK; 2 Centre for Neuroimaging Sciences, Institute of Psychiatry, Psychology and Neuroscience, King’s College London, London, UK; 3 Department of Basic and Clinical Neuroscience Institute of Psychiatry, Psychology and Neuroscience, King’s College London, London, UK; 4 MRC Centre for Neurodevelopmental Disorders, King’s College London, London, SE1 1UL UK; 5 Psychiatric Imaging Group, Faculty of Medicine, MRC London Institute of Medical Sciences (LMS), Imperial College London, London, UK

**Keywords:** Cannabinoid 1 receptor, CB1R agonists, CB1R antagonists, Cognition, Memory

## Abstract

**Rationale:**

While cannabis-based medicinal products have been shown to be effective for numerous neurological and psychiatric disorders, the evidence base regarding their adverse cognitive effects is poorly understood. The cannabinoid 1 receptor modulates memory performance via intracellular and extracellular mechanisms that alter synaptic transmission and plasticity. While previous literature has consistently shown that chronic cannabis users exhibit marked cognitive impairments, mixed findings have been reported in the context of placebo-controlled experimental trials. It is therefore unclear whether these compounds inherently alter cognitive processes or whether individuals who are genetically predisposed to use cannabis may have underlying cognitive deficits.

**Objective:**

We conducted a meta-analysis to investigate the effects of full and partial cannabinoid 1 receptor (CB1R) agonists, antagonists, and negative allosteric modulators on non-spatial and spatial memory.

**Methods:**

In accordance with the PRISMA guidelines, the EMBASE, MEDLINE, and PsycINFO databases were systematically searched for studies examining the effects of CB1R agonists, antagonists, and negative allosteric modulators on memory performance.

**Results:**

We systematically reviewed 195 studies investigating the effects of cannabinoid compounds on memory. In humans (*N* = 35 studies, comprising *N* = 782 subjects), delta-9-tetrahydrocannabinol (THC) (1.5–5 mg/kg) relative to placebo impaired performance on non-spatial memory tests, whereas only high THC doses (67 mg/kg) impaired spatial memory. Similarly, THC (0.2–4 mg/kg) significantly impaired visuospatial memory in monkeys and non-human primates (*N* = 8 studies, comprising *N* = 71 subjects). However, acute THC (0.002–10 mg/kg) had no effect on non-spatial (*N* = 6 studies, comprising 117 subjects; *g* = 1.72, 95% confidence interval (CI) − 0.18 to 3.63, *p* = 0.08) or spatial memory (9 studies, comprising 206 subjects; *g* = 0.75, 95% confidence interval (CI) − 1.09 to 2.58, *p* = 0.43). However, acute, full CB1R agonists significantly impaired non-spatial memory (*N* = 23 studies, 519 subjects; *g* = − 1.39, 95% CI − 2.72 to − 0.06, *p* = 0.03). By contrast, the chronic administration of CB1R agonists had no effect on non-spatial memory (*N* = 5 studies, comprising 146 subjects; *g* = − 0.05, 95% confidence interval (CI) − 1.32 to 1.22, *p* = 0.94). Moreover, the acute administration of CB1R antagonists had no effect on non-spatial memory in rodents (*N* = 9 studies, *N* = 149 subjects; *g* = 0.40, 95% CI − 0.11 to 0.92, *p* = 0.12).

**Conclusions:**

The acute administration of THC, partial CB1R agonist, significantly impaired non-spatial memory in humans, monkeys, and non-human primates but not rodents. However, full CB1R agonists significantly impaired non-spatial memory in a dose-dependent manner but CB1R antagonists had no effect on non-spatial memory in rodents. Moreover, chronic THC administration did not significantly impair spatial or non-spatial memory in rodents, and there is inconclusive evidence on this in humans. Our findings highlight species differences in the effects of cannabinoid compounds on memory.

**Electronic supplementary material:**

The online version of this article (10.1007/s00213-019-05283-3) contains supplementary material, which is available to authorized users.

## Introduction

Recent changes in legislation in many countries around the world, including the UK, Canada, and 30 states across the USA, have led to the widespread availability of cannabis-based medicinal products. Emerging evidence indicates that cannabis-based medicinal products may have analgesic (De Vita et al. 2018; Fitzcharles et al. 2016), antiemetic (Chang et al. 1979; Orr and McKernan 1981), antidyskinetic (Fox et al. 2002), antispasmodic (Zajicek et al. 2003), antiepileptic (Devinsky et al. 2014), and antipsychotic effects (McGuire et al. 2017; Boggs et al. 2018). However, the evidence base regarding the adverse cognitive effects of these cannabinoid compounds is unclear.

While the marijuana plant contains over 546 chemicals including over 104 cannabinoid compounds (ElSohly et al. 2016), the two most widely studied cannabinoids include delta-9-tetrahydrocannabinol (THC), a partial cannabinoid 1 receptor agonist (Huestis et al. 2001), and cannabidiol (CBD), a cannabinoid 1 receptor (CB1R) negative allosteric modulator (Morales et al. 2016). The concentration of THC is higher relative to CBD in street cannabis (THC:CBD ratio, 1:13) (ElSohly et al. 2016) and medical cannabis (THC:CBD ratio, 1:3) (Belendiuk et al. 2015). Since these two compounds have opposite pharmacological effects, the effects of THC are likely to outweigh the effects of CBD in the context of recreational or medicinal cannabis use.

Chronic cannabis users show impairments in memory encoding, storage, and retrieval (Solowij et al. 2011), and these deficits are greater if cannabis use commences prior to the age of 16 (Schuster et al. 2016). While some studies have reported that cannabis-induced memory impairments are no longer shown following abstinence (28 days) (Pope et al. 2001), other studies have shown that prior cannabis users continue to show marked memory impairments despite abstinence (28–60 days) (Thames et al. 2014; Schwartz et al. 1989; Schweinsburg et al. 2008). In line with these findings, cannabis use has also been found to impair memory in first episode psychosis (Núñez et al. 2016) and multiple sclerosis (Patel and Feinstein 2017), whereas cannabidiol has been shown to prevent the adverse effects of THC on memory (Englund et al. 2013a).

However, placebo-controlled experimental trials comparing the effects of THC relative to placebo on memory have reported discrepant findings, reporting no effects on memory (Ganon-Elazar and Akirav 2009; Geresu et al. 2016), memory enhancing (Amal et al. 2010; Bilkei-Gorzo et al. 2017), and memory-impairing effects (Yousefi et al. 2013; Santana et al. 2016; Goodman and Packard 2014). Similarly, while cannabidiol has been postulated to have cognitive enhancing effects (Englund et al. 2013b), the evidence regarding the therapeutic potential of these compounds is largely mixed (McGuire et al. 2017; Boggs et al. 2018; Rosenberg et al. 2017). Moreover, it is unclear if CB1R affinity, dose, treatment duration or treatment paradigms used may influence the effects of these compounds on memory.

Since THC is a partial CB1R agonist (Huestis et al. 2001) that has dose-dependent effects on memory (D’Souza et al. 2005), we aimed to investigate the effects of THC as well as compounds acting as agonists, antagonists, and negative allosteric modulators. We conducted a systematic review and meta-analysis of the acute and chronic effects of all full and partial CB1R agonists, CB1R antagonists, and CB1R negative allosteric modulators on spatial and non-spatial memory in humans, monkeys, non-human primates, rats, and mice. We predicted that the acute and chronic administration of full and partial CB1R agonists would induce spatial and non-spatial memory impairments, whereas CB1R antagonists and negative allosteric modulators would improve spatial and non-spatial memory performance.

## Method

### Search strategy

A single search was conducted for both animal and human studies. In accordance with the PRISMA guidelines, EMBASE, MEDLINE, PsycINFO, and PsycARTICLES databases were searched from 1950 to 1 September 2018 using the following search terms: (1) “cannabinoid 1 receptor agonist” OR “cannabinoid agonist” OR “cannabinoid receptor 1 antagonist” OR “cannabinoid antagonist” OR “CB1R negative allosteric modulator” OR “cannabis” OR “tetrahydrocannabinol” OR “anandamide” OR “WIN,55,212-2” OR “ACPA” OR “CP55940” OR “AM251” OR “SR161716A” OR “rimonabant” OR “cannabidiol” AND (2) “memory” OR “encoding” OR “recall” OR “retrieval.” The search criteria were registered on the international prospective register for systematic reviews.

### Selection criteria

The same criteria were applied for animal and human studies. General inclusion criteria for the systematic review and meta-analysis were (1) original research articles; (2) in vivo experimental methods; (3) comparison of drug relative to control (either placebo or vehicle); and (4) use of a memory paradigm (see supplementary materials 1 for full descriptions of memory paradigms). General exclusion criteria for the systematic review and meta-analysis were (1) review articles; (2) in vitro experimental methods; (3) failure to use a memory paradigm; (4) use of receptor knockout paradigms; (5) use of disease models; and (6) use of concurrent environmental manipulations (e.g. stress or food deprivation models). Studies were meta-analyzed together if they met the following criteria: use of the same (1) species; (2) pharmacological compound (e.g., CB1R agonist vs. antagonist); (3) intraperitoneal administration.

### Data extraction

In accordance with the PRISMA guidelines, the following variables were extracted from all animal and human studies: (1) authors; (2) year of publication; (3) sample characteristics (species, strain, sex, age, weight, and sample size); (4) drug characteristics (name, dose, route of drug administration, time point of drug administration); (5) memory paradigm; (6) results (mean and variance of memory performance in drug-treated group and control-treated group) (see supplementary materials). In cases where studies reported multiple drug doses, the highest available dose was selected for the primary analyses, but secondary dose-response relationships were examined where sufficient data were available. In this context, acute administration was defined as the administration of 1 dose, sub-chronic was defined as the administration of 2–4 doses, and chronic administration was defined as the administration of 5 or more doses on consecutive days. Memory performance data were extracted from tables or graphs using the plot digitizer where necessary (WebPlotDigitizer 2018). In cases where it was unclear if datasets were independent, the manuscript reporting the largest dataset was selected. All datasets included in the meta-analysis were independent.

### Study sample and methodological characteristics

The study flow chart is shown in supplementary figure 1. The literature search identified 2679 records which were manually screened by two independent researchers. After removing duplicates (*N* = 185), conference abstracts (*N* = 183), review articles (*N* = 90), irrelevant records (*N* = 1784), and books (*N* = 1), 436 full-text records were screened for eligibility. An additional 241 records were excluded due to the simultaneous administration of more than one drug (*N* = 13); failure to use CB1R agonist, antagonist, or negative allosteric modulator (*N* = 153); failure to use a memory paradigm (*N* = 8); and use of a disease model (*N* = 67). In total, 195 articles were deemed suitable for inclusion in the systematic review including 38 human studies, 8 primate studies, and 149 rodent studies, and 60 of these studies were suitable for inclusion in the meta-analyses.

### Statistical analysis

The main outcome measure in our analysis was the summary effect size (Hedge’s *g* (Hedges and Olkin 1985)) for the difference in memory performance between control-treated and drug-treated groups. We performed separate meta-analyses for CB1R agonists, antagonists, and negative allosteric modulators. In each meta-analysis, we compared the active treatment against control and analyzed acute and chronic studies separately. CB1R agonists with different formulations including WIN,55,212-2; ACPA; CP55,940; and delta-9-tetrahydrocannabinol (THC) were grouped into a single analysis. However, we also investigated the effects of full agonists (WIN,55,212-2; ACPA; CP55,940; anandamide) and partial agonist THC relative to vehicle. All comparisons were conducted with the statistical programming language R Studio (version 3.3.2) using the “metafor” package. Standardized effect sizes (Hedges’ *g* using a 95% confidence interval and a significance level of *p* < 0.05 (two-tailed)) for individual studies were first estimated. An overall summary effect size was then calculated by entering these individual study effect sizes into a random effects meta-analytic model using restricted maximum likelihood estimation.

Meta-analysis was conducted if there were at least 3 studies that used the same species, drug, administration route, and memory paradigm. Consistent with previous research, the highest drug dose was selected from each study (Kokkinou et al. 2018). All datasets included in the meta-analysis were independent. If at least 5 studies were included in a meta-analysis, dose-response relationships were also investigated.

Between-study inconsistency was estimated using the *I*^2^ value (*I*^2^ < 50% indicates low to moderate inconsistency, whereas *I*^2^ > 50% indicates moderate to high inconsistency). Publication bias was assessed in cases where there were at least 5 available studies by visual inspection of a funnel plot and the use of the Egger’s test. In cases where publication bias was suspected, a trim-fill analysis was conducted. If at least 5 studies were included in a meta-analysis, leave-one-out sensitivity analyses were conducted to ensure that the results were not driven by a single study.

Since previous literature has shown age-dependent (Solowij et al. 2011) and dose-dependent effects (D’Souza et al. 2005), meta-regressions were conducted to examine the effect of age and dose of the pharmacological compound on memory performance. We then compared subgroups by fitting a meta-regression model where the subgroup category acted as the moderating variable of interest. If this showed statistically significant differences between subgroups, a random-effects meta-analysis was conducted for each subgroup. The subgroups that we investigated were species, age, sex, compound (full, partial agonists), dose, paradigm, and drug administration timing (drug given before vs. after paradigm training), separately for non-spatial and spatial memory.

## Results

All datasets included in the meta-analyses were independent.

We report meta-analytic findings investigating the effects of cannabinoid compounds on spatial and non-spatial memory in rodents, followed by separate analyses studies using mice (see supplementary tables 1–3) and rats (see supplementary tables 4–6). Non-human primate or monkey studies are summarized in supplementary tables 7–9, and human studies are summarized in supplementary tables 10–12. See supplementary materials for descriptions of memory paradigms.

### Acute effects of CB1R agonists on non-spatial memory

**Fig. 1:**
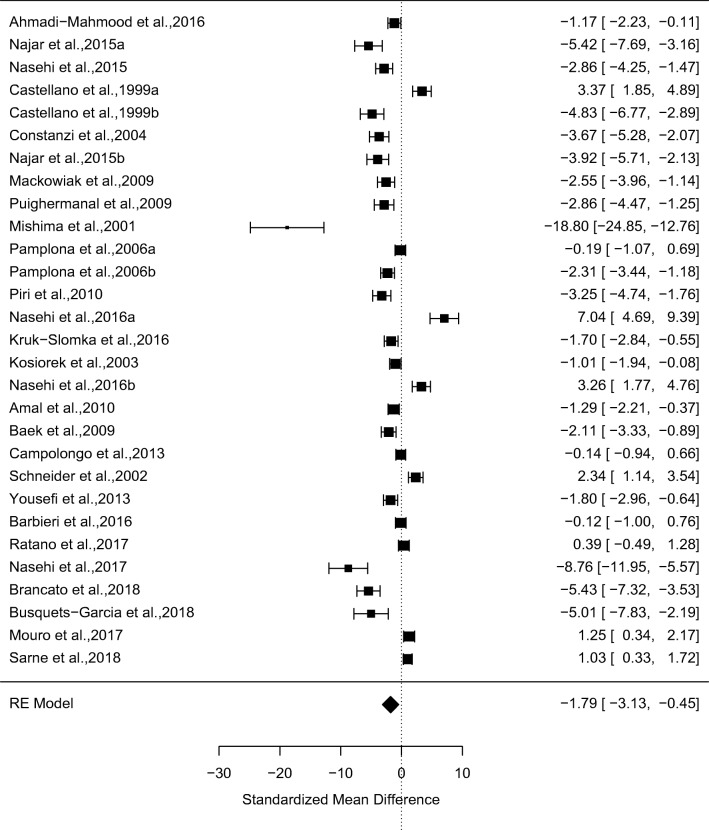
Forest plot from a meta-analysis of the acute effects of CB1R agonists relative to vehicle on non-spatial memory performance in rodents (*g* = − 1.79, 95% confidence interval (CI) − 3.13 to − 0.45, *p* = 0.009)

In a meta-analysis of 29 studies, CB1R agonists (*N* = 261) relative to vehicle (*N* = 258) significantly impaired memory performance on non-spatial memory paradigms (*g* = − 1.79, 95% confidence interval (CI) − 3.13 to − 0.45, *p* = 0.009) (see Fig. 1 and supplementary Figure 1 for funnel plot). There were high levels of between-study inconsistency (*I*^2^ = 97.50, *p* < 0.001). Egger’s test indicated that there was evidence of publication bias (*z* = 4.84, *p* < 0.0001), and a trim-fill analysis indicated that there were no missing studies. The results remained significant in all cases of the leave-one-out sensitivity analysis.

#### Effect of species

The magnitude of the effect of CB1R agonists vs. vehicle on non-spatial memory did not significantly vary with rodent species (*z* = 1.13, *p* = 0.26).

#### Effect of sex

The magnitude of the effect of CB1R agonists vs. vehicle on non-spatial memory did not significantly vary with sex (z = 0.79, *p* = 0.43).

#### Effect of paradigm

The magnitude of the effect of paradigm on CB1R agonists vs. vehicle on non-spatial memory did not significantly vary with the use of the inhibitory avoidance paradigm or the novel object paradigm (*z* = 0.86, *p* = 0.40).

#### Effect of drug administration timing

The magnitude of the effect of CB1R agonists vs. vehicle on non-spatial memory did not vary with drug administration timing (*z* = − 0.06, *p* = 0.95).

#### Effect of dose

The magnitude of the effect of CB1R agonists vs. vehicle on non-spatial memory inversely varied with dose (*z* = − 2.10, *p* = 0.04).

#### Effect of age

The magnitude of the effect of CB1R agonists vs. vehicle on non-spatial memory did not vary with age (*z* = 0.63, *p* = 0.53) or if rodents were adolescents (postnatal day (PND) < 65) or adults (PND > 65) (*z* = 1.19, *p* = 0.24).

#### Effect of drug

A meta-analysis of 23 studies indicated that full CB1R agonists relative to vehicle significantly impaired non-spatial memory (*g* = − 1.39, 95% confidence interval (CI) − 2.72 to − 0.06, *p* = 0.03 (see supplementary figures 2–3 for forest and funnel plots, respectively). The magnitude of the effect of CB1R agonists vs. vehicle on non-spatial memory did not vary with the compound used (*z* = − 0.21, *p* = 0.84). By contrast, a meta-analysis of 6 studies showed that partial CB1R agonist, delta-9-tetrahydrocannabinol, had no effects on memory performance (6 studies, *g* = − 4.04, 95% confidence interval (CI) − 9.30 to 1.17, *p* = 0.13) (see supplementary figures 4–5 for forest and funnel plots, respectively).

### Acute effects of CB1R antagonists on non-spatial memory

**Fig. 2:**
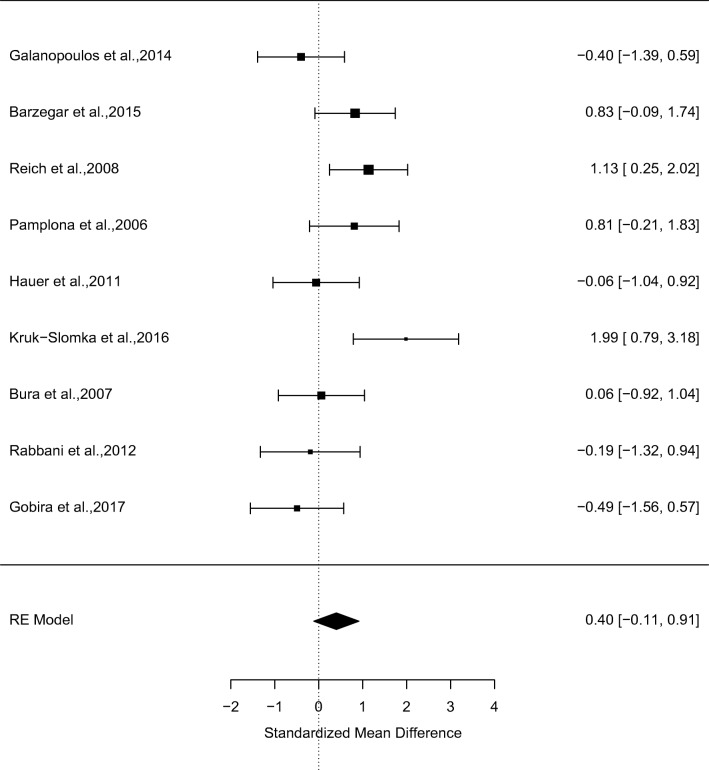
Forest plot from a meta-analysis of the effects of CB1R antagonists relative to vehicle on non-spatial memory performance in rodents (*g* = 0.40, 95% confidence interval (CI) − 0.11 to 0.92, *p* = 0.12)

In a meta-analysis of 9 studies, CB1R antagonists (*N* = 73) relative to vehicle (*N* = 76) had no effects on non-spatial memory performance (*g* = 0.40, 95% confidence interval (CI) − 0.11 to 0.92, *p* = 0.12) (see Fig. 2 and supplementary figure 6). There were moderate levels of between-study inconsistency (*I*^2^ = 56.78%, *p* = 0.02). Egger’s test indicated that there was no evidence of publication bias (*z* = − 0.20, *p* = 0.84), and a trim-fill analysis indicated that there were no missing studies. Findings remained unchanged in a leave-one-out sensitivity analysis.

#### Effect of species

The magnitude of the effect of CB1R antagonists vs. vehicle on non-spatial memory did not vary with rodent species (*z* = − 1.10, *p* = 0.29).

#### Effect of sex

The magnitude of the effect of CB1R antagonists vs. vehicle on non-spatial memory did not vary with sex (*z* = 1.54, *p* = 0.12).

#### Effect of paradigm

The magnitude of the effect of CB1R antagonists vs. vehicle on non-spatial memory did not vary with paradigm (*z* = − 1.73, *p* = 0.08).

#### Effect of drug administration timing

The magnitude of the effect of CB1R antagonists vs. vehicle on non-spatial memory was greater following pre-training relative to post-training drug administration (*z* = − 2.30, *p* = 0.02).

#### Effects of dose

The magnitude of the effect of CB1R antagonists vs. vehicle on non-spatial memory did not vary with dose (*z* = 0.34, *p* = 0.73).

#### Effect of age

The magnitude of the effect of CB1R antagonists vs. vehicle on non-spatial memory did not vary with age (*z* = − 0.62, *p* = 0.53).

#### Effect of drug

The magnitude of the effect of CB1R antagonists vs. vehicle on non-spatial memory did not vary with the compound used (*z* = 0.11, *p* = 0.91).

### Acute effects of CB1R negative allosteric modulators on non-spatial memory

There were insufficient studies to investigate the effects of negative allosteric modulators on non-spatial memory.

### Acute effects of CB1R agonists on spatial memory

In a meta-analysis of 11 studies, CB1R agonists (*N* = 113) relative to vehicle (*N* = 113) did not significantly impair memory performance on spatial memory paradigms (*g* = 0.75, 95% confidence interval (CI) − 1.68 to 3.18, *p* = 0.55) (see supplementary figures 7–8 for forest and funnel plot, respectively). There were moderate-high levels of between-study inconsistency (*I*^2^ = 97.50, *p* < 0.001). Egger’s test indicated that there was evidence of publication bias (*z* = − 1.61, *p* = 0.11), and a trim-fill analysis indicated that there were no missing studies. The results remained unchanged in all cases of the leave-one-out sensitivity analysis.

#### Effect of species

Moreover, the magnitude of the effect of CB1R agonists vs. vehicle on spatial memory did not vary with rodent species (*z* = − 0.74, *p* = 0.50).

#### Effect of sex

The magnitude of the effect of CB1R agonists vs. vehicle on spatial memory was greater in females relative to males (*z* = − 2.20, *p* = 0.03). However, there were insufficient studies to conduct further subgroup analyses on this.

#### Effect of paradigm

The magnitude of the effect of CB1 agonists vs. vehicle on spatial memory did not vary with the paradigm used (*z* = 1.41, *p* = 0.16).

#### Effect of drug administration timing

The magnitude of the effect of CB1R agonists vs. vehicle on spatial memory did not vary with drug administration time point (*z* = 0.89, *p* = 0.38).

#### Effect of dose

The magnitude of the effect of CB1R agonists vs. vehicle on spatial memory did not vary with dose (*z* = 0.4, *p* = 0.69).

#### Effect of age

The magnitude of the effect of CB1R agonists vs. vehicle did not vary with age (*z* = 0.92, *p* = 0.36).

#### Effect of drug

While there were insufficient studies to investigate the effects of full CB1R on spatial memory. Findings remained unchanged when restricting the analysis to partial CB1R agonist, THC relative to vehicle (9 studies, *g* = 0.75, 95% confidence interval (CI) − 1.09 to 2.58, *p* = 0.43) (see supplementary figures 9–10 for forest and funnel plots, respectively).

### Acute effects of CB1R antagonists and negative allosteric modulators on spatial memory

There were insufficient studies to investigate the effects of cannabinoid 1 receptor antagonists and negative allosteric modulators on spatial memory.

### Moderator analyses

The magnitude of the acute effect of CB1R agonists vs. vehicle did not significantly vary depending on the type of memory investigated (non-spatial versus spatial) (*z* = 1.89, *p* = 0.06). However, the magnitude of the chronic effects of CB1R agonists vs. vehicle was greater for non-spatial memory relative to spatial memory (*z* = 2.40, *p* = 0.02). The magnitude of the acute effect of CB1R agonists vs. vehicle on non-spatial and spatial memory did not significantly vary depending on whether rodents were adolescents or adults (*z* = − 0.64, *p* = 0.52).

### Chronic effects of CB1R agonists on non-spatial memory

**Fig. 3:**
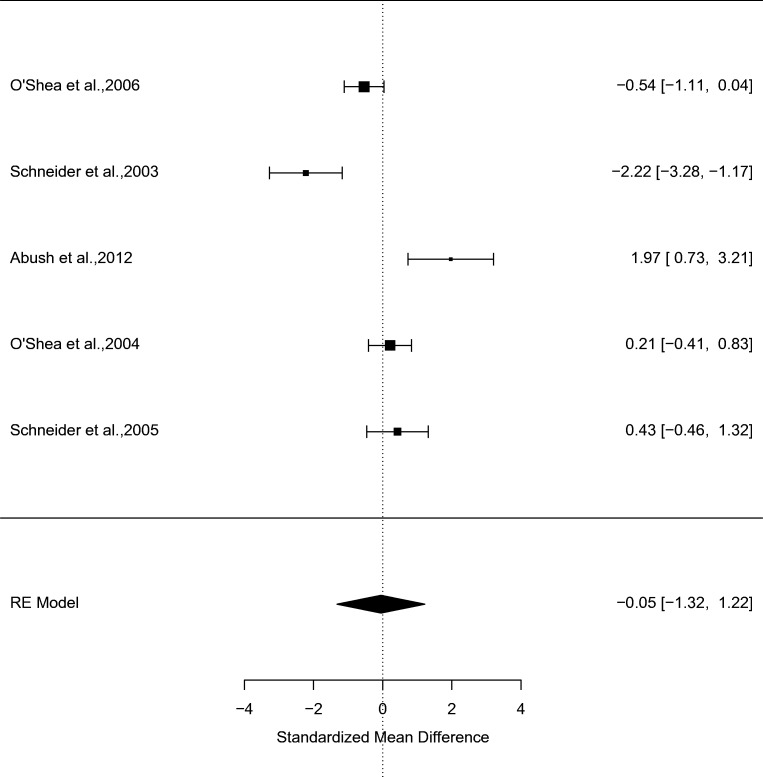
Forest plot from a meta-analysis of the chronic effects of CB1R agonists relative to vehicle on non-spatial memory performance in rodents (*g* = − 0.05, 95% confidence interval (CI), − 1.32 to 1.22, *p* = 0.94)

Our search identified 5 studies investigating the chronic effects of CB1R agonists (*N* = 53) relative to vehicle (*N* = 52) on non-spatial memory. Relative to vehicle, chronic administration of CB1R agonists did not significantly impair memory performance (*g* = − 0.05, 95% confidence interval (CI) − 1.32 to 1.22, *p* = 0.94) (see Fig. 3 and supplementary figure 11). There was no evidence of between-study inconsistency (*I*^2^ = 91.85%, *p* < 0.001). Egger’s test indicated that there was no evidence of publication bias (*z* = 0.42, *p* = 0.67), and a trim-fill analysis indicated that there were no missing studies.

#### Effect of species

Since all 5 studies were conducted in rats, no further sensitivity analyses were conducted.

#### Effect of sex

The magnitude of the effect of chronic CB1R agonists vs. vehicle on non-spatial memory did not vary with sex (*z* = − 0.97, *p* = 0.33).

#### Effect of paradigm

Since all 5 studies used the novel object paradigm, no further sensitivity analyses were conducted.

#### Effect of drug administration timing

The magnitude of the effect of chronic CB1R agonists vs. vehicle on non-spatial memory did not significantly vary with drug administration timing (*z* = − 1.92, *p* = 0.05).

#### Effect of dose

The magnitude of the effect of chronic CB1R agonists vs. vehicle on non-spatial memory did not significantly vary with dose (*z* = 0.20, *p* = 0.88).

#### Effect of age

The magnitude of the effect of chronic CB1R agonists relative to vehicle on non-spatial memory did not vary with age (*z* = 0.30, *p* = 0.78).

#### Effect of drug

In a meta-analysis of 5 studies, chronic CB1R agonists relative to vehicle did not significantly impair non-spatial memory (*N* = 5 studies, *N* = 146 subjects, *g* = − 0.05, 95% confidence interval (CI), − 1.32 to 1.22, *p* = 0.94). There were insufficient studies to investigate the effects of THC vs. vehicle on non-spatial memory.

### Chronic effects of CB1R agonists on spatial memory

Our search identified 5 studies investigating the chronic effects of CB1R agonists (*N* = 76) relative to vehicle (*N* = 70) on spatial memory, as determined by the Morris water maze (see supplementary figures 12–13 for forest and funnel plots, respectively). Relative to vehicle, chronic administration of CB1R agonists did not significantly impair memory performance (*g* = − 0.23, 95% confidence interval (CI) − 0.62 to 0.16, *p* = 0.24) (see supplementary figures 14–15 for forest and funnel plots, respectively). There was no evidence of between-study inconsistency (*I*^2^ = 0.0%, *p* < 0.0001). Egger’s test indicated that there was no evidence of publication bias (*z* = 0.91, *p* = 0.36), and a trim-fill analysis indicated that there were no missing studies.

#### Effect of species

The magnitude of the effect of chronic CB1R agonists vs. vehicle on spatial memory did not vary with species (*z* = − 0.50, *p* = 0.63).

#### Effect of sex

The magnitude of the effect of chronic CB1R agonists vs. vehicle on spatial memory did not vary with sex (*z* = − 1.20, *p* = 0.24).

#### Effect of paradigm

Since all 5 studies used the Morris water maze, no further sensitivity analyses were conducted.

#### Effect of drug administration timing

The magnitude of the effect of chronic CB1R agonists vs. vehicle on spatial memory did not vary with drug administration timing (*z* = − 0.97, *p* = 0.33).

#### Effect of dose

The magnitude of the effect of chronic CB1R agonists vs. vehicle on spatial memory did not vary with dose (*z* = 0.55, *p* = 0.58).

#### Effect of age

The magnitude of the effect of chronic CB1R agonists vs. vehicle on spatial memory did not vary with age (*z* = − 0.79, *p* = 0.43).

#### Effect of drug

There were insufficient studies to investigate the effects of full vs. partial CB1R agonists.

### Chronic effects of cannabinoid 1 receptor antagonists/negative allosteric modulators on spatial memory

There were no studies investigating the chronic effects of CB1R antagonists or negative allosteric modulators on spatial memory.

### The effects of cannabinoid compounds on memory in monkeys and non-human primates

See supplementary material tables 7–9 for a review of monkey and non-human primate studies. In monkeys and non-human primates, THC (0.2–4 mg/kg) significantly impaired memory performance on the delayed matching-to-sample task in 4 out of 5 studies. THC (0.2–0.3 mg/kg) also significantly impaired memory performance on the visual-spatial paired associates test in a single study.

### The effects of cannabinoid compounds on memory in humans

See supplementary material tables 10–12 for a review of human studies. THC (1.5–5 mg/kg) decreased immediate and delayed recall performance on the Hopkins verbal learning test in 4 out of 5 studies in the context of acute and sub-chronic administration. However, THC given chronically for 7 days (10–30 mg) had no effect on immediate or delayed recall on the Hopkins verbal learning test. Acute and sub-chronic THC (7.5–15 mg/kg) decreased emotional memory in 2 out of 2 studies. In 2 studies, acute THC (7.5 mg/kg) and acute CBD (32 mg) had no effect on memory extinction. Acute THC (0.018–0.036 mg/kg) decreased memory performance on the Rey Auditory Verbal Learning Test in 2 out of 2 studies. Acute rimonabant (20 mg/kg) had no effect on verbal learning on the Rey Auditory Verbal Learning Test in 2 out of 2 studies. Chronic (10 weeks) CBD (200 mg) improved word recall on the Rey Auditory Verbal Learning Test. In 3 studies, acute THC decreased spatial memory at 66.67 mg/kg, acute THC improved memory at 5 mg but had no effect on spatial memory at 2 or 3 mg/kg.

## Discussion

In line with our predictions, the acute administration of THC significantly impaired immediate and delayed recall, emotional memory, and verbal learning in humans, and visuospatial memory in non-human primates and monkeys. In contrast to our predictions, the acute and chronic administration of delta-9-tetrahydrocannabinol had no effect on spatial or non-spatial memory in rodents. Moreover, full CB1R agonists had no effect on spatial memory but CB1R agonists significantly impaired non-spatial memory in rodents.

In contrast to our predictions, CB1R agonists did not impair spatial or non-spatial memory when administered chronically, irrespective of age, sex, rodent species or drug administration timing relative to task training. This finding was not influenced by rodent species, sex, paradigm, drug administration timing, dose, age or the compound used. While further studies are needed to investigate the chronic effects of THC in humans, a single study found that THC (10–20 mg) had no effect on verbal memory performance in humans when administered chronically for 7 days (Mathai et al. 2018).

Interestingly, the detrimental effects of acute, full CB1R agonists on non-spatial memory were not influenced by rodent species, sex, age, paradigm, drug administration timing but impairments were inversely associated with dose. However, in contrast to our predictions, CB1R antagonists had no effect on non-spatial memory and this finding was not influenced by rodent species, sex, paradigm, dose, compound or age. However, the acute effects of CB1R antagonists on non-spatial memory were greater if the drug was administered prior to task training than after training.

## Strengths and limitations

A strength of the study was that we examined the effects of CB1R agonists, antagonists, and negative allosteric modulators on memory performance across a wide range of species using an array of spatial and non-spatial memory paradigms. Although this allowed us to quantitatively examine the consistency of the acute and chronic effects of CB1R agonists on memory in rodents, insufficient data was available to quantitatively investigate the acute or chronic effects of these compounds in humans, monkeys or non-human primates.

A limitation of the study was that we combined different drug doses which may potentially include effective and ineffective doses. However, in order to address this limitation, we investigated the dose-dependent effects of CB1R agonists and antagonists on memory performance. Moreover, although adolescents and adults were combined, we did not observe any age-dependent effects of CB1R agonists and antagonists on memory, and thus this is also unlikely to be a significant confound.

The high levels of between-study inconsistency observed may be linked to the combination of various pharmacological compounds with different binding profiles. However, we addressed this limitation by conducting sensitivity analyses of full and partial CB1R agonists. Another limitation of the study was that we combined mice and rat studies into a single meta-analysis. However, we aimed to overcome this limitation by conducting moderator analyses investigating species effects.

Another limitation of the study was that some meta-analyses included between 4 and 10 studies. As such, we cannot exclude the possibility of type II errors since these analyses may have been statistically underpowered to detect small group differences. While we found evidence of slight publication bias for meta-analyses of the acute effects of agonists on spatial and non-spatial memory, there was no evidence of publication bias for meta-analysis of acute antagonists or chronic agonists on spatial or non-spatial memory.

Previous literature has shown that THC briefly increases locomotor activity which is followed by a decrease in locomotor activity relative to baseline conditions (Bruijnzeel et al. 2016). As such, we cannot exclude the possibility that the locomotor effects of THC influenced memory performance in rodents. However, since we did not show evidence of impairments on spatial memory paradigms which involve locomotor function, locomotor effects of cannabinoids are unlikely to be a significant confound.

## Implications

Our finding that CB1R compounds selectively altered non-spatial memory but had no effects on spatial memory in rodents is consistent with previous literature indicating that spatial and non-spatial memory have distinct, dissociable neurobiological correlates (Park et al. 2016) and that brain lesions can selectively impair non-spatial memory in the absence of spatial memory deficits (Ravizza et al. 2006; De Renzi and Nichelli 1975) and vice versa (Rhodes et al. 2008; Backer Cave and Squire 1992; Hanley et al. 1991). However, our findings extend the existing literature by showing that the CB1R contributes to the mechanisms underlying non-spatial memory. However, future studies are needed to investigate this quantitatively in human, monkey or non-human primates.

Our finding that partial agonist THC significantly impaired non-spatial memory performance in humans on tasks involving verbal recall indicates that the endocannabinoid system may play a key role in the mechanisms underlying verbal memory. Previous literature has shown that cannabis users show non-spatial, verbal memory impairments with effect sizes ranging between *d* = 0.1–0.7 (Schoeler et al. 2016). However, in contrast to our predictions, THC did not significantly impair non-spatial memory in rodents. This discrepancy may be explained by species differences as well as methodological differences in the studies used across species.

In particular, if THC specifically impairs verbal memory in humans, this may not be seen in rodents due to differences in language capabilities. Species differences may also be linked to underlying differences in the endocannabinoid system, particularly the cannabinoid 1 receptor, that mediates the effects of these compounds (Huestis et al. 2001). Alternatively, these discrepant findings may relate to the use of intraperitoneal administration in rodents and intravenous administration in humans. However, since comparable THC peak plasma levels are reached following intravenous administration in humans (5 mg/kg) (Ohlsson et al. 1980) and following intraperitoneal administration in rats (3–10 mg/kg) (Nguyen et al. 2016), it is unlikely that species differences in drug bioavailability may explain this discrepant finding but further work is needed to investigate this.

It should also be recognized that there have been surprisingly few studies of THC effects on memory in rodents and, given the wide confidence interval in our meta-analysis, our analyses highlight the need for further studies of effects of both acute and chronic THC on memory. While we did not observe sex differences in the effects of CB1R agonists on non-spatial memory, we observed sex differences in the effects of CB1R agonists on spatial memory despite the fact that there were no significant effects of CB1R agonists on spatial memory.

This finding is consistent with previous work showing that humans show sex differences in CB1R availability (Park et al. 2016) as well as sex differences in the effects of THC on spatial memory (Ravizza et al. 2006).

Surprisingly, there were no differences in the effects of CB1R agonists vs. vehicle on memory between adults and adolescents. This finding is at odds with previous suggestions that adolescents may be more vulnerable to the effects of cannabinoids (Bossong and Niesink 2010). While no placebo-controlled studies have been conducted in human adolescents, THC vs. placebo has been found to significantly impair memory in adolescent monkeys (Backer Cave and Squire 1992). Moreover, further work is needed to investigate if low doses of THC may improve memory in old age in humans, as shown in rodents (Amal et al. 2010). In contrast to our predictions, CB1R antagonists failed to significantly enhance non-spatial memory and the evidence is largely mixed. Since we found that the effects of CB1R antagonists on non-spatial memory were greater when administered prior to task training, discrepant findings may be due to methodological differences in drug administration methods.

Despite differences in the acute vs. chronic effects of cannabinoid compounds on memory, these findings were not influenced by differences in age, sex, species or drug administration methods. Since few chronic studies have been conducted, further work is needed to investigate factors that may account for the different effects of acute vs. chronic drug administration. Our finding that the acute administration of CB1R agonists impairs memory, but chronic administration does not, may reflect underlying changes in the cannabinoid 1 receptor. Since chronic exposure to cannabinoids decreases the expression of extracellular cannabinoid receptors (Hsieh et al. 2002), the chronic administration of cannabinoids may have less dramatic downstream effects on memory due to the presence of cannabinoid 1 receptors. In line with this, chronic cannabis users exhibit fewer cannabinoids 1 receptors that normalize following abstinence (D’Souza et al. 2016).

While these findings are consistent with literature showing that cannabis-induced memory impairments are not shown following abstinence (28 days) (Pope et al. 2001), other studies have shown that prior cannabis users continue to show marked memory impairments despite abstinence (28–60 days) (Thames et al. 2014; Schwartz et al. 1989; Schweinsburg et al. 2008). Therefore, underlying genetic differences may predispose individuals to THC-induced cognitive impairments. In line with this, converging lines of evidence indicate that functional polymorphisms in the cannabinoid 1 receptor (CNR1) gene are associated with behavioral and functional measures of working memory performance in healthy volunteers. In particular, healthy volunteers exhibiting functional polymorphisms in the CNR1 gene (C carriers of rs1406977) show reduced BOLD responses in the dorsolateral prefrontal cortex during working memory performance in the absence of behavioral impairments in working memory (Taurisano et al. 2016), whereas healthy volunteers showing functional polymorphisms in the CNR1 gene (GG carriers of rs2180619) show reduced working memory performance and lower performance under higher working memory load demands (Ruiz-Contreras et al. 2014).

Future studies are also needed to investigate the effects of full CB1R agonists and antagonists on both spatial and non-spatial memory in humans. Future studies are needed to identify if the endocannabinoid system may modulate other aspects of memory such as episodic, semantic, and procedural memory. Our finding that chronic THC had no effect on spatial or non-spatial memory performance is consistent with findings from human literature showing that THC has no effect on memory performance when administered chronically (Mathai et al. 2018). However, future studies are needed to investigate the chronic effects of THC in placebo-controlled trials in order for this to be quantitatively investigated. Moreover, while there is preliminary evidence to suggest that negative allosteric modulators can enhance memory performance in humans when administered chronically, further studies are needed.

Our finding that the acute administration of full CB1R agonists impairs non-spatial memory performance in rodents is consistent with literature showing that CB1R activation inhibits LTP, a key physiological mechanism underlying memory formation (Navakkode and Korte 2014a). CB1R activation has been specifically shown to impair LTP by dysregulating the protein synthesis involved in the formation and degradation of synaptic connections (Navakkode and Korte 2014b). Our finding that CB1R activation impairs memory performance is also consistent with evidence that CB1R activation impairs memory by directly altering mitochondrial energy metabolism required for cellular activity (Huestis et al. 2001). The deleterious effects of CB1R activation on memory performance are mediated via CB1R-dependent modulation of soluble adenylyl cyclase (sAC), an enzyme that catalyzes the conversion of adenosine triphosphate (ATP), a vital source of energy, into cyclic adenosine monophosphate (cAMP), a second messenger involved in intracellular signaling cascades that activates protein kinase A (Morales et al. 2016).

## Conclusions

Our findings collectively show that the THC has species-specific effects on memory, impairing non-spatial memory in humans, monkeys, non-human primates but not rodents. Moreover, the chronic administration of CB1R agonists failed to impair non-spatial or spatial memory in rodents. In contrast to our predictions, acute CB1R agonists selectively impaired non-spatial, but not spatial memory, and the acute administration of CB1R antagonists had no effects on non-spatial memory in rodents. Future placebo-controlled studies in humans are needed to investigate the cognitive effects of chronic THC administration as well as the cognitive effects of low dose THC for cognitive impairment in old age.

## Electronic supplementary material

ESM 1 (DOCX 784 kb)
