# Supplementary material for: The effects of cannabinoid 1 receptor compounds on memory: a meta-analysis and systematic review across species
Source: Psychopharmacology (Berl). 2019 Jun 5;236(11):3257–70. doi: 10.1007/s00213-019-05283-3 (PMC6828623; doi:10.1007/s00213-019-05283-3)
Supplement: Supplementary file 1 — (DOCX 784 kb) [file 213_2019_5283_MOESM1_ESM.docx]

**Supplementary materials**

**Supplementary table 1. Mice studies: sample characteristics**

| **Source** | **Journal** | **Age** | **Sex** | **Weight or BMI** | **N** | **Species** | **Strain** |
| --- | --- | --- | --- | --- | --- | --- | --- |
| Amal et al., (2010) | Brain Research Bulletin | 8-12 weeks old | male | 20-40 g | 6-10 per group | mice | ICR |
| Barbieri et al., (2016) | Neuropsychopharmacology | 4-6 weeks old | male | 25-30 g | 10 per group | mice | ICR |
| Bilkei-Gorzo et al., (2017) | Nature Medicine | 2, 12, 18 months |  | not reported | 7-15 per group | mice | C57BL/6J |
| Boucher et al., (2009) | Behavioural Pharmacology | adult. Age not reported | male | 23-28 g | 12 per group | mice | C57BL/6J |
| Castellano et al., (1997) | Behavioural Pharmacology | 8 weeks | male | 28-30 g (8 weeks) | 8 per group | mice | CD1 |
| Castellano et al., (1999) | Behavioural Pharmacology | 8 weeks | male | 20-23 g | 6-8 per group | mice | C57BL/6 (C57) and DBA |
| Constanzi et al., (2003) | Neurobiology of Learning and Memory | not reported | male | 30 g | 8 per group | mice | CD1 |
| Constanzi et al., (2004) | Neurobiology of Learning and Memory | 8 weeks | male | 25-30 g | 8 per group | mice | CD1 |
| Da Silva et al., (2002) | Progress in Neuro- Pharmacology and Biological Psychiatry | not reported | male | 25-30 g | 8-12 per group | mice | Albino |
| Geresu et al., (2016) | Brain Research | 6-8 weeks old | male | 25-35g | 6 per group | mice | albino |
| Gleason et al., (2012) | Translational Psychiatry | adolescent (30-35 PND) and adult (63-70 PND) | not reported | not reported | 10 per group | mice | C57BL6 |
| Hayakawak et al., (2008) | Brain Research | not reported | male | 25-35g | 5 per group | mice | ddY |
| Jafari-Sorbet et al., (2017) | Pharmacology, Biochemistry and Behaviour | not reported | male | 30-35g | 10 per group | mice | NMRI |
| Kasten et al., (2017) | Pharmacology, Biochemistry and Behaviour | adolescents (3 weeks) and adults (8 weeks) | not reported | not reported | 10 per group | mice | inbred C57Bl/6J and DBA/2J |
| Laviollete et al., (2006) | The Journal of Neuroscience | not reported | not reported | 275-350g | 6 per group | mice | Sprague-Dawley |
| Mohammadi et al., (2015) | Behavioural Brain research | not reported | male | 30-35g | 8 per group | mice | NMRI |
| Mouro et al., (2017) | Neuopsychopharmacology | 8-12 weeks old | male | not reported | 8-12 per group | mice | C57BL6 |
| Nasehi et al., (2015) | Behavioural Brain research 286:245-363 | not reported | male | 25-30g | 10 per group | mice | NMRI |
| Nasehi et al., (2016) | Brain Research 1636(142-151) | not reported | male | 28-33 g | 8 per group | mice | NMRI |
| Nasehi et al., (2016) | Journal of Psychopharmacology 30(9):936-944 | not reported | male | 28-32g | 10 per group | mice | NMRI |
| Nasehi et al., (2016) | Psychopharmacology 233(2):213-224 | not reported | male | 28-32 g | 10 per group | mice | NMRI |
| Nasehi et al., (2017) | psychopharmacology 234(20): 317-3128 | not reported | male | 30-35g | 8 per group | mice | NMRI |
| Nasehi et al., (2018) | Psychopharmacology 32(8), 932-942 | 6-8 weeks | male | 28-33g | 8 per group | mice | NMRI |
| Nasehi et al., (2017) | Brain research bulletin 131, 85-92 | not reported | male | 28-33g | 8 per group | mice | NMRI |
| Nasehi et al., (2016) | Psychopharmacology, 233, 213-224 | not reported | male | 28-32g | 8 per group | mice | NMRI |
| Puighermanal et al., (2009) | Nature Neuroscience | not reported | male | 25-30g | 4-6 per group | mice | CD1 |
| Sarne et al., (2018) | Neurobiology of Aging | young (2 months) and old age (24 months) | female | not reported | old (12-18 per group) | mice | not reported |
| Yousefi et al., (2013) | Neurobiology of Learning and Memory | not reported | male | 30-35g | 8 per group | mice | NMRI |
| Zarrindast et al., (2010) | Neuroscience | not reported | male | 22-25 g | 10 per group | mice | NMRI |

ICR=Institute of Cancer research; PND=post-natal day; g=gram

**Supplementary table 2. Mice studies: drug administration methods**

| **Source** | **Type of drug** | **Name of drug** | **Drug administration method** | **Dose** | **Type of administration: acute/chronic** | **Drug administration relative to training*** | **Drug administration relative to test** |
| --- | --- | --- | --- | --- | --- | --- | --- |
| Amal et al., (2010) | agonist | THC | intraperitoneal | 0.002 and 10 mg/kg | Acute (single dose) | post-training | drug given 5 weeks before test |
| Barbieri et al., (2016) | agonist | JWH-018, THC & AM251 | intraperitoneal | JWH-018 (0.01, 0.1, 0.3 & 1 mg/kg) & THC (0.1, 0.3, 1, 3 mg/kg) | Acute (single dose) | post-trianing | 2 hours and 24 hours before test |
| Bilkei-Gorzo et al., (2017) | agonist | THC | intraperitoneal | 3 mg/kg per day | chronic (28 doses over days) | post-training | 5 or 14 days before test |
| Boucher et al., (2009) | agonist | THC | intraperitoneal | 1 mg/kg | chronic (13 doses over days) | post-training | 30 mins before test |
| Castellano et al., (1997) | agonist | AEA | intraperitoneal | 1.5, 3, 6 mg/kg | Acute (single dose) | post-training | 24 hours after test |
| Castellano et al., (1999) | agonist | AEA | intraperitoneal | 1.5, 3, 6 mg/kg | Acute (single dose) | post-training | 24 hours after test |
| Constanzi et al., (2003) | agonist | AEA | intraperitoneal | 1.5, 3, 6 mg/kg | Acute (single dose) | post-training | 24 hours before test |
| Constanzi et al., (2004) | agonist | AEA | intraperitoneal | 1.5, 3, 6 mg/kg | Acute (single dose) | post-training | 24 hours before test |
| Da Silva et al., (2002) | agonist | THC | intraperitoneal | 4, 6, 8 mg/kg | Acute (single dose) | pre-training | 30 mins before test |
| Geresu et al., (2016) | agonist | WIN55,212-2 | intraperitoneal | 1 mg/kg | Acute (single dose) | pre-training | 20 mins before test |
| Gleason et al., (2012) | agonist | WIN55,212-2 | intraperitoneal | 2 mg/kg | sub-chronic (3-5 doses over 3-5 days or 10 doses over days) | pre-training | either 57 (adult) 90 days (adolescent) before test |
| Hayakawak et al., (2008) | agonist | THC, CBD | intraperitoneal | THC (1, 3 & 6 mg/kg) & cannabidiol (1, 3, 10, 25, 50 mg/kg) | Acute (single dose) | pre-training | 1 hour before test |
| Jafari-Sorbet et al., (2017) | agonist | ACPA | hippocampus | 1 & 2 ng/rodent | Acute (single dose) | post-training | 24 hours before test or immediately before test |
| Kasten et al., (2017) | agonist | THC | intraperitoneal | 10 mg/kg | chronic (N=6 doses, one every 72 hours) | post-training | 4 weeks before test |
| Laviollete et al., (2006) | agonist | WIN 55,212-2 | ingralimbic region of mPFC | 5, 25, 50 ng | Acute (single dose) | pre-training | 24 hours before test |
| Mohammadi et al., (2015) | agonist | ACPA | amygdala | 0.05, 0.01, .05 mg/kg | Acute (single dose) | post-training | 24 hours before test |
| Mouro et al., (2017) | agonist | WIN55,212-2 | intraperitoneal | 1 mg/kg | Acute (single dose) | post-training | 24 hours before test |
| Nasehi et al., (2015) | agonist | ACPA | hippocampus | 2, 5, 10 mg/kg | Acute (single dose) | pre-training | 24 hours before test |
| Nasehi et al., (2016) | agonist | ACPA | amygdala | 0.01, 0.05, 0.1, 0.5 mg/kg per rodent | Acute (single dose) | post-training | 24 hours before test |
| Nasehi et al., (2016) | agonist | ACPA | intraperitoneal | 0.01, 0.05, 0.1 mg/kg | Acute (single dose) | pre-training | 24 hours before test |
| Nasehi et al., (2016) | agonist | ACPA | intraperitoneal | 0.01, 0.02, 0.01 | Acute (single dose) | pre-training | 24 hours before test |
| Nasehi et al., (2017) | agonist | ACPA | hippocampus | 0.02 mg/kg | Acute (single dose) | post-training | 24 hours before test |
| Nasehi et al., (2018) | agonist | ACPA | intraperitoneal | 0.005, 0.05, 0.5 mg/kg | Acute (single dose) | pre-training | 24 hours before test |
| Nasehi et al., (2017) | agonist | ACPA | intraperitoneal | 0.005, 0.1, 0.5 mg/kg | Acute (single dose) | pre-training | 24 hours before test |
| Nasehi et al., (2016) | agonist | ACPA | amygdala | 0.01, 0.02, 0.1 mg/kg | Acute (single dose) | pre-training | 24 hours before test |
| Puighermanal et al., (2009) | agonist | THC | intraperitoneal | 10 mg/kg | Acute (single dose) | post-training | 24 hours before test |
| Sarne et al., (2018) | agonist | THC | intraperitoneal | 0.002 mg/kg | Acute (single dose) | post training | 3-5 weeks before test |
| Yousefi et al., (2013) | agonist | ACPA | intraperitoneal | 0.005, 0.01, 0.02 mg/kg | Acute (single dose) | post-training | 24 hours before test |
| Zarrindast et al., (2010) | agonist | WIN 55,212-2 | hippocampus | 1 ug/rodent | Acute (single dose) | post-training and pre-training | 24 hours before test |

*pre-training=drug given before training; post-training=drug given after training; ACPA= N-(Cyclopropyl)-5Z,8Z,11Z,14Z-eicosatetraenamide; THC=delta-9-tetrahydrocannabinol, CBD=cannabidiol, AM251= N-(Piperidin-1-yl)-5-(4-iodophenyl)-1-(2,4-dichlorophenyl)-4-methyl-1H-pyrazole-3-carboxamide; CP55,940= (-)-cis-3-[2-Hydroxy-4-(1,1-dimethylheptyl)phenyl]-trans-4-(3-hydroxypropyl)cyclohexanol; HU-210= (6aR)-trans-3-(1,1-Dimethylheptyl)-6a,7,10,10a-tetrahydro-1-hydroxy-6,6-dimethyl-6H-dibenzo[b,d]pyran-9-methanol; anandamide= N-(2-Hydroxyethyl)-5Z,8Z,11Z,14Z-eicosatetraenamide; SR141716A= N-(Piperidin-1-yl)-5-(4-chlorophenyl)-1-(2,4-dichlorophenyl)-4-methyl-1H-pyrazole-3-carboxamide hydrochloride; (R)-(+)-[2,3-Dihydro-5-methyl-3-(4-morpholinylmethyl)pyrrolo[1,2,3-de]-1,4-benzoxazin-6-yl]-1-naphthalenylmethanone mesylate; mg=milligram; kg=kilogram; ug=microgram; JWH-018= 1-pentyl-3-(1-naphthoyl)indole; mins=minutes

**Supplementary table 3. Mice studies: key findings**

| **Source** | **Type of drug** | **Name of drug** | **Type of administration: acute/chronic** | **Design** | **Memory paradigm** | **Results** |
| --- | --- | --- | --- | --- | --- | --- |
| Amal et al., (2010) | agonist | THC | acute | cross-sectional | Object recognition task | ↓ memory performance at 0.002 and 10 mg/kg |
| Barbieri et al., (2016) | agonist | JWH-018, THC & AM251 | acute | cross-sectional | novel object | ↓ memory performance with JWH-018 at doses 0.01, 0.1, 0.3 & 1 mg/kg; ↓ memory performance with THC at doses 3 mg/kg |
| Bilkei-Gorzo et al., (2017) | agonist | THC | chronic (28 days) | cross-sectional | Morris water maze | ↓ memory performance in young mice (2 months); No effect in mature mice (12 months)). THC ↑ memory in old mice (18 months) |
| Boucher et al., (2009) | agonist | THC | chronic (13 days) | cross-sectional | Morris water maze | ↓ memory performance |
| Castellano et al., (1997) | agonist | AEA | acute | cross sectional | inhibitory avoidance | ↓ memory performance at 3 and 6 mg/kg |
| Castellano et al., (1999) | agonist | AEA | acute | cross sectional | inhibitory avoidance | DBA: ↓ memory performance at 3 and 6 mg/kg; C57: ↑ memory at 3 and 6 mg/kg |
| Constanzi et al., (2003) | agonist | AEA | acute | cross sectional | inhibitory avoidance | ↓ memory performance at 3 and 6 mg/kg |
| Constanzi et al., (2004) | agonist | AEA | acute | cross sectional | inhibitory avoidance | ↓ memory performance at 3 and 6 mg/kg |
| Da Silva et al., (2002) | agonist | THC | acute | cross-sectional | morris water maze | ↓ memory performance at 8 mg/kg |
| Geresu et al., (2016) | agonist | WIN55,212-2 | acute | cross-sectional | Y maze | no effect |
| Gleason et al., (2012) | agonist | WIN55,212-2 | sub-chronic (3-5 or 10 days) | cross-sectinal | inhibitory avoidance | adolescent treatment ↑ FAAH & MGL reflecting greater ecs uptake/degeneration. |
| Hayakawak et al., (2008) | agonist | THC, CBD | acute | cross-sectional | eight arm radial maze | ↓ memory performance at 3 & 6 mg/kg |
| Jafari-Sorbet et al., (2017) | agonist | ACPA | acute | cross-sectional | inhibitory avoidance | ↓ memory performance. Drug administration before test reversed ACPA amnesia |
| Kasten et al., (2017) | agonist | THC | chronic (N=6 total, one every 72 hours) | cross-sectional | Novel object | No effects of age or treatment on memory |
| Laviollete et al., (2006) | agonist | WIN 55,212-2 | acute | cross sectional | olfactory fear conditioning | ↓ performance at dose 50 ng |
| Mohammadi et al., (2015) | agonist | ACPA | acute | cross-sectional | novel object | ↓ performance at 0.05 mg/kg |
| Mouro et al., (2017) | agonist | WIN55,212-2 | acute | cross-sectional | novel object | ↓ memory performance |
| Nasehi et al., (2015) | agonist | ACPA | acute | cross-sectional | inhibitory avoidance | ↓ memory performance at 10 mg/kg |
| Nasehi et al., (2016) | agonist | ACPA | acute | cross-sectional | inhibitory avoidance | ↓ performance |
| Nasehi et al., (2016) | agonist | ACPA | acute | cross-sectional | inhibitory avoidance | ↓ memory performance at 0.01 ug/rodent |
| Nasehi et al., (2016) | agonist | ACPA | acute | cross-sectional | inhibitory avoidance | ↓ memory performance at dose 0.1 mg/kg |
| Nasehi et al., (2017) | agonist | ACPA | acute | cross-sectional | novel object | ↓ memory performance at 0.02 ug/rodent |
| Nasehi et al., (2018) | agonist | ACPA | acute | cross-over | inhibitory avoidance | ↓ memory performance at 0.5 mg/kg |
| Nasehi et al., (2017) | agonist | ACPA | acute | cross-over | inhibitory avoidance | pre-training administration ↑ memory performance 0.1 and 0.5 mg/kg |
| Nasehi et al., (2016) | agonist | ACPA | acute | cross-sectional | inhibitory avoidance | ↓ memory performance at 0.1 mg/kg |
| Puighermanal et al., (2009) | agonist | THC | acute | cross-sectional | novel object & inhibitory avoidance | ↓ memory performance |
| Sarne et al., (2018) | agonist | THC | acute | cross-sectional | object recognition, Y maze and Morris water maze | Object recognition & Y maze: ↑ memory (THC old age group preferred novel vs familiar object). Vehicle old group - no difference between novel vs. familiar object. Young vehicle group preferred novel object vs. familiar. |
| Yousefi et al., (2013) | agonist | ACPA | acute | cross-sectional | novel object | ↓ memory performance |
| Zarrindast et al., (2010) | agonist | WIN 55,212-2 | acute | cross-sectional | inhibitory avoidance | ↓ performance at doses 1 ug/rodent |

ACPA= N-(Cyclopropyl)-5Z,8Z,11Z,14Z-eicosatetraenamide; THC=delta-9-tetrahydrocannabinol, CBD=cannabidiol, AM251= N-(Piperidin-1-yl)-5-(4-iodophenyl)-1-(2,4-dichlorophenyl)-4-methyl-1H-pyrazole-3-carboxamide; CP55,940= (-)-cis-3-[2-Hydroxy-4-(1,1-dimethylheptyl)phenyl]-trans-4-(3-hydroxypropyl)cyclohexanol; HU-210= (6aR)-trans-3-(1,1-Dimethylheptyl)-6a,7,10,10a-tetrahydro-1-hydroxy-6,6-dimethyl-6H-dibenzo[b,d]pyran-9-methanol; anandamide= N-(2-Hydroxyethyl)-5Z,8Z,11Z,14Z-eicosatetraenamide; SR141716A= N-(Piperidin-1-yl)-5-(4-chlorophenyl)-1-(2,4-dichlorophenyl)-4-methyl-1H-pyrazole-3-carboxamide hydrochloride; (R)-(+)-[2,3-Dihydro-5-methyl-3-(4-morpholinylmethyl)pyrrolo[1,2,3-de]-1,4-benzoxazin-6-yl]-1-naphthalenylmethanone mesylate; mg=milligram; kg=kilogram; ug=microgram; JWH-018= 1-pentyl-3-(1-naphthoyl)indole

**Supplementary table 4. Rat studies: sample characteristics**

| **Source** | **Journal** | **Age** | **Sex** | **Weight or BMI** | **N** | **Species** | **Strain** |
| --- | --- | --- | --- | --- | --- | --- | --- |
| Abbousi et al., (2014) | Pharmacology, Biochemistry and Behaviour | adolescent (27-30 PND) and adult (54-57 PND) | male | adolescent (M=82kg) and adult (M=261kg) | adolescent (10 per group); adults (11 per group) | rats | Wistar |
| Abush et al., (2012) | Plos one | adolescent (45 days old) | male | 200g | vehicle=8, WIN=7 | rats | Sprague-Dawley |
| Ahmadi-Mahmood et al., (2016) | Neuroscience | adult. Age not reported | male | 250-290 g | 8 per group | rats | Sprague-Dawley |
| Ashton et al., (2008) | Pharmacology, Biochemistry and Behaviour | not reported | not reported | not reported | 5-6 per group | rats | not reported |
| Baek et al., (2009) | Neuroscience letters | 6 months | male | 500-600g | vehicle=7, 1mgkg=7; 3mg/kg=7; 5 mg/kg=8 | rats | Wistar |
| Barros et al., (2004) | Brain Research | 2.5 months | male | 220-280 g | 8 per group | rats | Wistar |
| Braida et al., (2000) | Learning and memory | not reported | male | 250-300 g | 10 per group | rats | Wistar |
| Beiravand et al., (2016) | Journal of Psychopharmacology | 2.5 months | male | 250-300 g | 8 per group | rats | Wistar |
| Campolongo et al., (2013) | Neuropsychopharmacology | adult. Age not reported. | male | 250-450g | 12 per group | rats | Sprague-Dawley |
| Carlini et al., (1965) | Psychopharmacologia | 90 days | male | not reported |  | rats | Wistar |
| Cha et al., (2006) | Pharmacology, Biochemistry and Behaviour | adolescent (30-32 PND) and adult (65-70 PND) | male | not reported | 8 per group | rats | Sprague-Dawley |
| Cha et al., (2007) | Behavioural Pharmacology | adolescent (30 PND) and adult (70 PND) | male and female | not reported | 8 per group | rats | Sprague-Dawley |
| Clarke et al., (2008) | Neurobiology of Learning and Memory | 3 months | male | 250-280g | 9 per group | rats | Wistar |
| De Melo et al., (2005) | Psychopharmacology | not reported | male | 250-300g | 11 per group | rats | Wistar |
| Egashira et al., (2002) | Brain Research | 7 weeks | male | 200-250g | vehicle=14, thc=6 | rats | Wistar |
| Egashira et al., (2002b) | European Journal of Pharmacology | 7 weeks | male | 200-250g | vehivle=13, thc=13 | rats | Wistar |
| Essman et al., (1984) | Psychological Reports | not reported | male | 375g | 12 per group | rats | Sprague-Dawley |
| Fadda et al., (2004) | Neuropsychopharmacology | not reported | male | 250-300g | 10 per group | rats | hooded Lister |
| Fitoussi et al., (2018) | European Journal of Neuroscience | not reported | male | 400-500g | 8 per group | rats | Sprague-Dawley |
| Ferrari et al., (1999) | Pharmacology, Biochemistry and Behaviour | not reported | male | 230-250g | 8 per group | rats | Wistar Hannover |
| Ganon et al., (2009) | The Journal of Neuroscience | 60 days old | male | 250-300g | 12-13 per group | rats | Sprague-Dawley |
| Goodman et al., (2014) | Neuroscience | adult. Age not reported. | male | 325-425g | peripheral (9-10 per group), central (6-8 per group) | rats | Long Evans |
| Ghaderi et al., (2016) | Progress in Neuro- Pharmacology and Biological Psychiatry | not reported | male | 200-220g | 7 per group | rats | Wistar |
| Ghasemdeh et al., (2017) | European Journal of Pharmacology | not reported | male | 200-220g | 7 per group | rats | Wistar |
| Ghiasvand et al., (2011) | Behavioural brain research | adult. Age not reported. | male | 220-270g | 8 per group | rats | Wistar |
| Han et al., (2000) | European Journal of Pharmacology | 120 days | male | not reported | 7 per group | rats | Sprague-Dawley |
| Hernandez et al., (2000) | Journal of Physiology and Biochemistry | 1 day | male | not reported | 11 per group | rats | Wistar |
| Imam et al., (2017) | Nigerian Journal of Physiological Sciences | adult. Age not reported | not reported | M=200g | 6 per group | rats | Wistar |
| Inui et al., (2004) | Neurotoxicity research | not reported | male | 200-250g | vehicle=10; THC=9 | rats | Wistar |
| Kevin et al., (2017) | Journnal of Psychopharmacology | PND 69-72 | not reported | 130-178g | 16 per group | rats | Wistar |
| Kirschman et al., (2017) | Frontiers in Behavioural Neuroscience | PND 34-53 | female | not reported | 8 per group | rats | Sprague-Dawley |
| Mallet et al., (1996) | Behavioural Pharmacology | not reported | male | 200-250g | 15-16 per group | rats | Wistar |
| Miller et al., (1973) | Behavioural Biology | 140-160 PND | male | not reported | 42 per group | rats | Hooded |
| Mackowiak et al., (2009) | Neuroscience | 60 days old | male | 200-250g | 7-10 per group | rats | Wistar |
| Mishima et al., (2001) | Japanese Journal of Pharmacology | not reported | male | 200-250g | 9=vehicle, 10-=thc | rats | Wistar |
| Mishima et al., (2002) | Life Sciences | not reported | male | 200-250g | 10 per group | rats | Wistar |
| Molina-Holgado et al., (1995) | Physiology and Behaviour | not reported | male | 150-220g | 8 per group | rats | Wistar |
| Nadaei et al., (2016) | Behavioural Brain research | adult. Age not reported. | male | M=200g | 7 per group | rats | Wistar |
| Murillo-Rodriguez et al., (1998) | Brain Research | not reported | male | 250-250g | 6 per group | rats | Wistar |
| Najar et al., (2015) | Journal of Psychopharmacology | 3-4 months | male | 200-220g | 7 per group | rats | Wistar |
| Nakamura et al., (1991) | Drug and Alcohol Dependence | 2 months | male | not reported (age 2 months) | 9 per group | rats | Wistar |
| Nava et al., (2001) | Neuropsychopharmacology | not reported | male | 200-250g | 5 per group | rats | Sprague-Dawley |
| O'Shea et al., (2004) | Psychopharmacology | adolescent (30 days) and adult (18, and 56 day old) | female | not reported | 20 per group | rats | Wistar |
| O'Shea et al., (2006) | Psychopharmacology | perinatal (4 days), adolescent (30 days), 56 days (young adult) | male | perinatal (9-12g), adolescent (77-123g), adult (269-348g) | 24 per group | rats | Wistar |
| Ottani et al., (2000) | Pharmacy and Pharmacology Communications | not reported | male | 230-250g | 8 per group | rats | Wistar |
| Pamplona et al., (2006) | Neuroscience letters | 3 months | male | weight not reported - 3 months | 7-8 per group | rats | Wistar |
| Pamplona et al., (2006) | Psychopharmacology | 3 months | male | weight not reported - 3 months | 7-10 per group | rats | Wistar |
| Pamplona et al., (2008) | Neurobiology of Learning and Memory | 3 months | male | weight not reported - 3 months | 9 per group | rats | Wistar |
| Pedroza-Llinas et al., (2013) | Behavioural Brain Research | not reported | male | 250-300g | AEA 56 μM (*n* = 10), 5.6 mM (*n* = 11), 11.4 mM (*n* = 8), and 56 mM (*n* = 4) groups were different from both vehicle (*n* = 9) and intact groups. | rats | Wistar |
| Piri et al., (2011) | Achives of Iranian Medicine | 4 months | male | 220-270g (4 months) | 8 per group | rats | Wistar |
| Quinn et al., (2008) | Neuropsychopharmacology | adolescent (PND 28+) and adult (PND 60+) | male | adolescent (M=76.2g) and adult (M=284g) | 12 per group | rats | Wistar |
| Raseki et al., (2014) | Behavioural brain research | adult. Age not reported. | male | 250-290g | 8 per group | rats | Wistar |
| Ratano et al., (2017) | Frontiers in Pharmacology | not reported | male | 350-450 g | 11-13 per group | rats | Sprague-Dawley |
| Robinson et al., (2010) | Behavioural Brain Research | not reported | male | 250-350g | 9 per group | rats | Lister Hooded |
| Sandler et al., (2017) | Plos computational biology | not reported | male | not reported | 6 total | rats | Long Evans |
| Santana et al., (2016) | Neurobiology of Learning and Memory | not reported | male | 270-320 g | 8 per group | rats | Sprague-Dawley |
| Schneider et al., (2002) | Behavioural Pharmacology | 25-30 days old | male | 250-300g | test=9; control=8 | rats | Wistar |
| Schneider et al., (2003) | Neuropsychopharmacology | adolescent (PND 40-60), adult (PND 70) | male and female | not reported | 9-14 per group | rats | Wistar Hannover |
| Schneider et al., (2005) | Behavioural Pharmacology | prepubertal period (PND 15-40) | male and female | M=81g | 9-11 per group | rats | Wistar Hannover |
| Schneider et al., (2008) | Addiction Biology | Adolescent (PND 40-65) | male and female | not reproted | 8-17 per group | rats | Wistar Hannover |
| Segev et al., (2011) | Learning and memory | 60 days old | male | 250-300g | 6-8 per group | rats | Sprague-Dawley |
| Stern et al., (2015) | European Neuropsychopharmacology | 14-16 weeks | male | not reported | 7-8 per group | rats | Wistar |
| Tan et al., (2011) | Journal of Neuroscience | not reported | male | 350-400g | drug =7, vehicle=6 | rats | Sprague-Dawley |
| Wise et al., (2009) | Neuropsychopharmacology | not reported | male | not reported | 9 per group | rats | Sprague-Dawley |
| Wegener et al., (2008) | Psychopharmacology | adult. Age not reported. | male | 200-250g | 6-8 per group | rats | Sprague-Dawley |
| Yim et al., (2008) | Neuroscience | not reported | male | 225-275g | 11 per group | rats | Long Evans |
| Zarrindast et al., (2011) | Neuroscience Research | not reported | male | 220-250g | 8 per group | rats | Wistar |
| Zarrindast et al., (2012) | Neuroscience Research | adult. Age not reported. | male | 220-270 g | 8 per group | rats | Wistar |

G=gram; PND=post-natal day

**Supplementary table 5. Rat studies: drug administration methods**

| **Source** | **Type of drug** | **Name of drug** | **Drug administrration method** | **Dose** | **Type of administration: acute/chronic** | **Drug administration relative to training (pre-training=drug given before training; post-training=drug given after training)** | **Drug administration relative to test** |
| --- | --- | --- | --- | --- | --- | --- | --- |
| Abbousi et al., (2014) | agonist | WIN 55,212-2 | intraperitoneal | 1 mg/kg | chronic (20 days) followed by 20 day washout | post-training | 20 days before test |
| Abush et al., (2012) | agonist | WIN 55,212-2 | intraperitoneal | 1.2 mg/kg | chronic (14 days) following by testing after either 1 day or 10 days | pre-training | test 24 h or 10 days after withdrawal |
| Ahmadi-Mahmood et al., (2016) | agonist | ACPA | mPFC | 0.001, 0.01, 0.1 and 0.5 ug/rat | acute | post-training | 24 hours before test |
| Ashton et al., (2008) | agonist | THC | intraperitoneal | 15 mg/kg | chronic (6 days) | post-training | not reported |
| Baek et al., (2009) | agonist | WIN 55,212-2 | intraperitoneal | 1, 3, 5 mg/kg | acute | post-training | 30 mins before test |
| Barros et al., (2004) | agonist | Anandamide | hippocampus | 100 uM (0.5 ug/side) | acute | pre-training | 24 hours before test |
| Braida et al., (2000) | agonist | CP55940 | intraperitoneal | .175, 0.2, 0.3 mg/kg | acute | post-training | 20 mins before test |
| Beiravand et al., (2016) | agonist | ACPA | mPFC | 0.05,0.05 and 0.5 μg/rodent | acute | pre-training | before training and 24 hours before test |
| Campolongo et al., (2013) | agonist | WIN 55,212-2 | intraperitoneal | 0.1, 0.3 and 1 mg/kg | acute | post-training | 1 hour before test |
| Carlini et al., (1965) | agonist | Cannabis Sativa | intraperitoneal | 10 mg/kg | acute | post-training | 3 mins before or 30 seconds after test |
| Cha et al., (2006) | agonist | THC | intraperitoneal | 2.5,5,10 mg/kg | acute (5 days) & chronic (21 days) | pre-training | 30 mins before training |
| Cha et al., (2007) | agonist | THC | intraperitoneal | 5 mg/kg | acute (5 days) & chronic (21 days) | pre-training | 30 mins before test |
| Clarke et al., (2008) | agonist | WIN 55,212-2 | hippocampus | 1, 2.5, 10 nmol/per rodent | acute | post training | 24 hours before test |
| De Melo et al., (2005) | agonist | THC | intraperitoneal and mPFC | IP (0.32, 1, 1,8 mg/kg) and IC mPFC (32, 100, 180 ug) | acute | post training | IP (30 mins before test), IC (5 mins before test) |
| Egashira et al., (2002) | agonist | THC | various brain regions including hippocampus, frontal cortex etc. | 6 mg/kg | acute | pre-training | 1 hour before test |
| Egashira et al., (2002b) | agonist | THC | intraperitoneal | 6 mg/kg | acute | pre-training | 1 hour before test |
| Essman et al., (1984) | agonist | marijuana | inhalation | 42 puffs within 15 mins | acute | post-training | 24 hours before test |
| Fadda et al., (2004) | agonist | THC | intraperitoneal | 0.5, 2, 5 mg/kg (THC) and 0.5, 5, 10 and 50 mg/kg (CBD). | acute | post-training | 30 mins before test |
| Fitoussi et al., (2018) | agonist | THC | nucleus accumbens | 50, 100 ng | acute | post-training | 24 hours before test |
| Ferrari et al., (1999) | agonist | HU210 | intraperitoneal | HU210 (25, 50, 100 ug/kg) | sub-chronic (4 days) | pre-training | 24 hours, 3 days and 7 days |
| Ganon et al., (2009) | agonist | WIN55,212-2 | amygdala | 0.5 ug per side | acute | pre and post-traning | 20 mins before conditioning or 20 mins before memory extinction |
| Goodman et al., (2014) | agonist | WIN55,212-2 | intraperitoneal | 1 or 3 mg/kg | acute | post-training | 24 hours before test |
| Ghaderi et al., (2016) | agonist | ACPA | hippocampus | 0.5, 2, 4 ng/rat | acute | pre-training | 24 hours before test |
| Ghasemdeh et al., (2017) | agonist | ACPA | hippocampus | 0.5, 0.75, 1 ng/rat | acute | post-training | 30 mins before test |
| Ghiasvand et al., (2011) | agonist | WIN 55,212-2 | amgdala | 0.01, 0.05,0.1 and 0.25 μg/rat | acute | post training | 24 hours before test |
| Han et al., (2000) | agonist | WIN55,212-2 | amygdala and medial prefrontal cortex | 0.5 ug | acute | post-training | 40 mins before test |
| Hernandez et al., (2000) | agonist | THC | intraperitoneal | 5 mg/kg | acute | post-training | 30 mins before test |
| Imam et al., (2017) | agonist | THC | intraperitoneal | 20 mg | chronic (7 days) | not reported | not reported |
| Inui et al., (2004) | agonist | THC | intraperitoneal | 6 mg/kg | acute | pre-training | 60 mins before test |
| Kevin et al., (2017) | agonist | THC | intraperitoneal | 6 mg/kg then 20 mg/kg | chronic - 12 doses (6 doses of 1 mg/kg then 6 doses of 5 mg/kg) | post-training | 2 weeks before test |
| Kirschman et al., (2017) | agonist | WIN55,212-2 | intraperitoneal | 0.2 mg/kg or 1.2 mg/kg | chronic (20 days) | pre-training | 24 hours before test |
| Mallet et al., (1996) | agonist | AEA & THC | intraperitoneal & hippocampal | AEA (0.0, 0.25, 0.5 1 and 2 mg/kg) & THC (0.5, 1, 2, 4 mg/kg) | chronic (4 days) | post-training | 5 mins before test |
| Miller et al., (1973) | agonist | THC | intraperitoneal | 5 mg/kg | acute | pre-training | 24 hours before test |
| Mackowiak et al., (2009) | agonist | HU-210 & AM251 | intraperitoneal | 0.1 mg/kg HU-210 & AM251 3 mg/kg | acute | post-training | 24 hours before test |
| Mishima et al., (2001) | agonist | THC | intraperitoneal | 10 mg/kg | acute | pre-training (post training would be repeated administration) | 1 hour before test |
| Mishima et al., (2002) | agonist | THC | intraperitoneal | 6 mg/kg | acute | post-training | 1 hour before test |
| Molina-Holgado et al., (1995) | agonist | THC | intraperitoneal | 5 mg/kg | acute | post-training | 30 mins before test |
| Nadaei et al., (2016) | agonist | ACPA | amygdala | 1, 3, 4 ng/rat | acute | post-training | 24 hours before test |
| Murillo-Rodriguez et al., (1998) | agonist | oleamide | intraperitoneal | 3.6 nmol | acute | post-training | either 15 mins of 24 hours before test |
| Najar et al., (2015) | agonist | ACPA | medial septum | 0.0002, 0.002, 0.02 ug/rat | acute | post-training | 24 hours before test |
| Nakamura et al., (1991) | agonist | THC | intraperitoneal | 1.25 mg/kg | acute | post-training | 30 mins before test |
| Nava et al., (2001) | agonist | THC | intraperitoneal | 2.5, 5 mg/kg | acute | post-training | 20 mins before test |
| O'Shea et al., (2004) | agonist | CP55940 | intraperitoneal | 150 (3 days), 200 (8 days), 300 ug/kg (10 days) | chronic (21 days) | pre-training | 21 days before test |
| O'Shea et al., (2006) | agonist | CP55940 | intraperitoneal | 0.15, 0.20, 0.30 (7 days per dose) | chronic (21 days) | post-training | 21 days before test |
| Ottani et al., (2000) | agonist | HU-210 | intraperitoneal | 25, 50, 100 ug/kg | sub-chronic (once daily for 4 days) | pre-training | 60 mins before test |
| Pamplona et al., (2006) | agonist | WIN 55,212-2 | intraperitoneal | 0.25, 1.25, 2.5 and 5 mg/kg | acute | pre-training | 24 hours before test |
| Pamplona et al., (2006) | agonist | WIN 55,212-2 | intraperitoneal | 0.2, 1 and 2 mg/kg | acute | pre-training | 24 hours before test |
| Pamplona et al., (2008) | agonist | WIN 55,212-2 | intraperitoneal | 0.25 mg/kg | acute | post training | 20 mins before extinction |
| Pedroza-Llinas et al., (2013) | agonist | AEA | nucleus accumbens | 56 uM, 5.6 mM, 56 mM /per rodent | acute | post training | 24 hours before test |
| Piri et al., (2011) | agonist | WIN 55,212-2 | intraperitoneal | 0.25 & 0.5 μg/rat | acute | pre- and post- | 24 hours before test |
| Quinn et al., (2008) | agonist | THC | intraperitoneal | 5 mg/kg | sub-chronic (2 days) | post-training | 12 days before test |
| Raseki et al., (2014) | agonist | ACPA & AM251 | intra-NAC | 0.15, 0.3, 3 ng/side (ACPA) & 0.3, 3, 30 ng/side (AM251) | acute | post-training | 24 hours before test |
| Ratano et al., (2017) | agonist | WIN 55,212-2 | intraperitoneal | 0.3, 1, 3 mg/kg | acute | post-training | 48 hours before test |
| Robinson et al., (2010) | agonist | WIN55,212-2, AM281, SR141716 | intraperitoneal | 1, 3 mg/kg (WIN55,212-2), 1 mg/kg (WIN55,212-3), 1 mg/kg WIN55,212-2 + 0.5 mg/kg AM281, 1 mg/kg WIN55,212-2 + 3 mg/kg Rimonabant | sub-chronic (4 days) | pre-training | 30 mins before test |
| Sandler et al., (2017) | agonist | THC | intraperitoneal | 1 mg/kg | acute | post-training | not reported |
| Santana et al., (2016) | agonist | CP55940 | hippocampus & infra-limbic cortex | 1, 5, 10 μg/per rodent | acute | post training | 48 hours or 7 days before test |
| Schneider et al., (2002) | agonist | WIN 55,212-2 | intraperitoneal | 0.6 & 1.2 mg/kg | acute | pre-training | 10 mins before test |
| Schneider et al., (2003) | agonist | WIN55,212-2 | intraperitoneal | 1.2 mg/kg | chronic (20 injections - 10 daily and 5 twice daily) | post-training | 10, 15, 20, 55 days before test |
| Schneider et al., (2005) | agonist | WIN55,212-2 | intraperitoneal | 1.2 mg/kg | chronic (20 injections - 10 daily and 5 twice daily) | not reported | 30 mins before test |
| Schneider et al., (2008) | agonist | WIN55,212-2 | intraperitoneal | 1.2 mg/kg | chronic (20 injections - 10 daily and 5 twice daily) | post-training | 24 hours or 15 days before test |
| Segev et al., (2011) | agonist | WIN55,212-2 and AM251 | amygdala & ventral subiculum | 0.5 ug/side (WIN) 0.3 ng/side (AM251) | acute | post-training | 30 mins before test |
| Stern et al., (2015) | agonist | THC | intraperitoneal | 0.1, 0.3, 1.0, 10 mg/kg | acute | post-training | 24 hours before test |
| Tan et al., (2011) | agonist | AM251 | amygdala | 2.5, 25, 50, 500 | acute | pre- and post-training | immediately before test |
| Wise et al., (2009) | agonist | CP55940 | hippocampus | 0.5 ul/side | acute | post-training | 20 mins before test |
| Wegener et al., (2008) | agonist | WIN55,212-2 | intraperitoneal & nucleus accumbens shell, VTA, mPFC, hippocampus | peripheraL (1.2 mg/kg) & central (5 μg/0.3 ul) | acute | post-training | 10 mins before test |
| Yim et al., (2008) | agonist | WIN55,212-2 | intraperitoneal | 1, 3 mg/kg | acute | post-training | 1 or 4 weeks before test |
| Zarrindast et al., (2011) | agonist | WIN 55,212-2 | hippocampus | 0.1, 0.25 and 0.5 μg/rat | acute | post-training | 24 hours before test |
| Zarrindast et al., (2012) | agonist | WIN 55,212-2 | hippocampus | 0.001, 0.05, 0.01, 0.25 ug/rat | acute | post-training | 24 hours before test |

ACPA= N-(Cyclopropyl)-5Z,8Z,11Z,14Z-eicosatetraenamide; THC=delta-9-tetrahydrocannabinol, CBD=cannabidiol, AM251= N-(Piperidin-1-yl)-5-(4-iodophenyl)-1-(2,4-dichlorophenyl)-4-methyl-1H-pyrazole-3-carboxamide; CP55,940= (-)-cis-3-[2-Hydroxy-4-(1,1-dimethylheptyl)phenyl]-trans-4-(3-hydroxypropyl)cyclohexanol; HU-210= (6aR)-trans-3-(1,1-Dimethylheptyl)-6a,7,10,10a-tetrahydro-1-hydroxy-6,6-dimethyl-6H-dibenzo[b,d]pyran-9-methanol; anandamide= N-(2-Hydroxyethyl)-5Z,8Z,11Z,14Z-eicosatetraenamide; SR141716A= N-(Piperidin-1-yl)-5-(4-chlorophenyl)-1-(2,4-dichlorophenyl)-4-methyl-1H-pyrazole-3-carboxamide hydrochloride; (R)-(+)-[2,3-Dihydro-5-methyl-3-(4-morpholinylmethyl)pyrrolo[1,2,3-de]-1,4-benzoxazin-6-yl]-1-naphthalenylmethanone mesylate; mg=milligram; kg=kilogram; ug=microgram; JWH-018= 1-pentyl-3-(1-naphthoyl)indole; ug=microgram; g=gram; kg=kilogram; mins=minutes

**Supplementary table 6. Rat studies: key findings**

| **Source** | **Type of drug** | **Name of drug** | **Drug administration method** | **Design** | **Memory paradigm** | **Results** |
| --- | --- | --- | --- | --- | --- | --- |
| Abbousi et al., (2014) | agonist | WIN 55,212-2 | intraperitoneal | cross-sectional | Morris water maze | ↓ memory performance in adolescents but not adults following chronic treatment (1 mg/kg for 20 days) and drug washout (20 days) |
| Abush et al., (2012) | agonist | WIN 55,212-2 | intraperitoneal | cross-sectional | Morris water maze and novel object | ↓ memory performance after 24h withdrawal but 10 days of withdrawal |
| Ahmadi-Mahmood et al., (2016) | agonist | ACPA | medial prefrontal cortex | cross-sectional | inhibitory avoidance | ↓ memory performance at 0.1 and 0.5 ug/rat |
| Ashton et al., (2008) | agonist | THC | intraperitoneal | cross-sectional | auditory inhibitory avoidance | ↑ memory performance (prevented extinction) |
| Baek et al., (2009) | agonist | WIN 55,212-2 | intraperitoneal | cross-sectional | novel object | ↓ memory performance at 5 mg/kg |
| Barros et al., (2004) | agonist | Anandamide | hippocampus | cross-sectional | inhibitory avoidance | ↓ memory performance at 100 uM |
| Braida et al., (2000) | agonist | CP55940 | intraperitoneal | cross-sectional | radial maze | agonist ↓memory at doses .2 and .3 mg/kg |
| Beiravand et al., (2016) | agonist | ACPA | medial prefrontal cortex | cross-sectional | inhibitory avoidance | ↓ performance at doses 0.05 and 0.5 ug/rodent |
| Campolongo et al., (2013) | agonist | WIN 55,212-2 | intraperitoneal | cross-sectional | novel object | ↓ memory performance at 0.3 mg/kg when no habituation given but improved memory when habituation training given. |
| Carlini et al., (1965) | agonist | Cannabis Sativa | intraperitoneal | cross-sectional | Lashey III alley maze | ↑ memory performance when drug given before task. No effect on memory when given after task. |
| Cha et al., (2006) | agonist | THC | intraperitoneal | cross sectional | Morris water maze | Acute: ↓ memory after acute (5 days) in adolescents (5 mg/kg) but not adults at any dose. Chronic: no effects of age or chronic treatment on memory |
| Cha et al., (2007) | agonist | THC | intraperitoneal | cross sectional | Morris water maze | Acute: female: ↓memory performance after 1-2 days at 5 mg/kg not 3-5 days. No effect in males. Chronic: no effect in males or females. |
| Clarke et al., (2008) | agonist | WIN 55,212-2 | hippocampus | cross sectional | novel object | ↓ performance at doses 1 and 2.5 nmol |
| De Melo et al., (2005) | agonist | THC | intraperitoneal and medial prefrontal cortex | cross-sectional | radial arm maze | ↓ memory performance both ip and ic |
| Egashira et al., (2002) | agonist | THC | various brain regions including hippocampus, frontal cortex etc. |  | eight arm radial maze | ↓ memory performance |
| Egashira et al., (2002b) | agonist | THC | intraperitoneal | cross-sectional | eight arm radial maze | ↓ memory performance |
| Essman et al., (1984) | agonist | marijuana | inhalation | cross-sectional | inhibitory avoidance | ↓ memory performance |
| Fadda et al., (2004) | agonist | THC | intraperitoneal | cross-over | Water maze | THC ↓ memory performance. CBD-rich extracts had no effect on memory performance |
| Fitoussi et al., (2018) | agonist | THC | nucleus accumbens | cross-sectional | olfactory fear conditioning | ↑ memory performance |
| Ferrari et al., (1999) | agonist | HU210 | intraperitoneal | cross-sectional | Morris water maze | no effect on memory at end of treatment or following abstinence (3/7 days later) |
| Ganon et al., (2009) | agonist | WIN55,212-2 | amygdala | cross-sectional | memory extinction | no effect |
| Goodman et al., (2014) | agonist | WIN55,212-2 | intraperitoneal | cross-sectional | Water maze | ↓ memory (peripheral 3 mg/kg dose and intra- dorsolateral striatum 200 ng per side) |
| Ghaderi et al., (2016) | agonist | ACPA | hippocampus | cross-sectional | inhibitory avoidance | ↓ memory performance at doses 0.5, 2 and 4 ng/rat |
| Ghasemdeh et al., (2017) | agonist | ACPA | hippocampus | cross-sectional | inhibitory avoidance | ↓ memory performance at dose 1 ng/rodent |
| Ghiasvand et al., (2011) | agonist | WIN 55,212-2 | amygdala | cross sectional | inhibitory avoidance | ↓ performance at dose 0.1 and 0.25 ug/rodent |
| Han et al., (2000) | agonist | WIN55,212-2 | amygdala and medial prefrontal cortex | cross-sectional | fear conditioning | agonist during adolescence ↓ memory in adulthood |
| Hernandez et al., (2000) | agonist | THC | intraperitoneal | cross-sectional | radial arm maze | ↓ memory performance at 5 mg/kg |
| Imam et al., (2017) | agonist | THC | intraperitoneal | cross-sectional | Y maze | ↓ memory performance at 20 mg |
| Inui et al., (2004) | agonist | THC | intraperitoneal | cross-sectional | eight arm radial maze | ↓ memory performance at 6 mg/kg |
| Kevin et al., (2017) | agonist | THC | intraperitoneal | cross-over | Novel object | ↓ memory after 60 mins but not 2 minutes |
| Kirschman et al., (2017) | agonist | WIN55,212-2 | intraperitoneal | cross-sectional | DMTS | No effect of low or high doses on memory |
| Mallet et al., (1996) | agonist | AEA & THC | intraperitoneal & hippocampal | cross-sectional | non-match to position task | ↓memory (THC 4 mg/kg or AEA 2 mg/kg) |
| Miller et al., (1973) | agonist | THC | intraperitoneal | cross-sectional | inhibitory avoidance | ↓ memory performance |
| Mackowiak et al., (2009) | agonist | HU-210 & AM251 | intraperitoneal | cross-sectional | inhibitory avoidance | ↓ memory performance & AM251 had no effect |
| Mishima et al., (2001) | agonist | THC | intraperitoneal | cross-sectional | inhibitory avoidance | ↓ memory performance at 10 mg/kg |
| Mishima et al., (2002) | agonist | THC | intraperitoneal | cross-sectional | 8-arm radial maze | ↓ memory performance |
| Molina-Holgado et al., (1995) | agonist | THC | intraperitoneal | cross-sectional | 8-arm radial maze | ↓ memory performance |
| Nadaei et al., (2016) | agonist | ACPA | amygdala | cross-over | inhibitory avoidance task | No effect on memory |
| Murillo-Rodriguez et al., (1998) | agonist | oleamide | intraperitoneal | cross-sectional | memory extinction | ↓ memory performance |
| Najar et al., (2015) | agonist | ACPA | medial septum | cross-sectional | inhibitory avoidance task | ↓ memory performance at 0.02 ug/rodent |
| Nakamura et al., (1991) | agonist | THC | intraperitoneal | cross-sectional | radial 8-arm maze | ↓ memory performance |
| Nava et al., (2001) | agonist | THC | intraperitoneal | cross-over | t maze | ↓ memory at 2.5 and 5 mg/kg after 20 and 60 mins |
| O'Shea et al., (2004) | agonist | CP55940 | intraperitoneal | cross-sectional | object recognition | ↓ memory in adolescent but not adult rats. |
| O'Shea et al., (2006) | agonist | CP55940 | intraperitoneal | cross-sectional | object recognition | agonist impaired memory at all ages |
| Ottani et al., (2000) | agonist | HU-210 | intraperitoneal | cross-sectional | Morris water maze | No effect at 25 ug/kg. Agonist impaired memory at 50 and 100 ug/kg on sessions 4-7 not 1-3. |
| Pamplona et al., (2006) | agonist | WIN 55,212-2 | intraperitoneal | cross-sectional | inhibitory avoidance | ↓ memory performance at doses 2.5 and 5 |
| Pamplona et al., (2006) | agonist | WIN 55,212-2 | intraperitoneal | cross-sectional | inhibitory avoidance | ↓ memory performance at dose 0.25 mg/kg only |
| Pamplona et al., (2008) | agonist | WIN 55,212-2 | intraperitoneal | cross-sectional | memory extinction | ↓ memory performance at 0.25 mg/kg |
| Pedroza-Llinas et al., (2013) | agonist | AEA | nucleus accumbens | cross-sectional | inhibitory avoidance | ↓ performance at all doses |
| Piri et al., (2011) | agonist | WIN 55,212-2 | intraperitoneal | cross-sectional | inhibitory avoidance | ↓ memory performance at doses 0.25 and 0.5 |
| Quinn et al., (2008) | agonist | THC | intraperitoneal | cross-sectional | novel object recognition | ↓ memory performance in adolescents. No effect of agonist vs vehicle in adults. |
| Raseki et al., (2014) | agonist | ACPA & AM251 | Nucleus accumbens | cross-sectional | inhibitory avoidance | ↓ memory performance at 3ng/side. No effect of AM251 |
| Ratano et al., (2017) | agonist | WIN 55,212-2 | intraperitoneal | cross-sectional | inhibitory avoidance | ↓ memory performance at 1 mg/kg |
| Robinson et al., (2010) | agonist | WIN55,212-2, AM281, SR141716 | intraperitoneal | cross-sectional | Perspex water maze | agonist alone ↓memory at 1 mg/kg. Agonist combined with antagonist (either AM 0.5 mg/kg or Rimonabant 3 mg/kg) impaired memory |
| Sandler et al., (2017) | agonist | THC | intraperitoneal | cross-over | DNMS | ↓ performance at 1 mg/kg |
| Santana et al., (2016) | agonist | CP55940 | hippocampus & infra-limbic cortex | cross-sectional | inhibitory avoidance | ↓ performance at 5 ug |
| Schneider et al., (2002) | agonist | WIN 55,212-2 | intraperitoneal | cross-sectional | object recognition | ↓ performance at doses 0.6 and 1.2 mg/kg |
| Schneider et al., (2003) | agonist | WIN55,212-2 | intraperitoneal | cross-sectional | object recognition | Adult: agonist had no effect on object recognition. Adolescent: agonist ↓ object recognition in adulthood. |
| Schneider et al., (2005) | agonist | WIN55,212-2 | intraperitoneal | cross-sectional | object recognition | No effect on object recognition |
| Schneider et al., (2008) | agonist | WIN55,212-2 | intraperitoneal | cross-over | object recognition | Adolescent: agonist ↓ post-training memory performance, Adult: no effect of agonist on memory performance |
| Segev et al., (2011) | agonist | WIN55,212-2 and AM251 | amygdala & ventral subiculum | cross-sectional | memory extinction | agonist into amygdala ↓ memory vs vehicle. Agonist + antagonist blocked effects on memory |
| Stern et al., (2015) | agonist | THC | intraperitoneal | cross-over | fear conditioning | THC ↑ memory (decreased % freezing) at doses 0.3-10 mg/kg 24 hours after drug. No effect 48 hours after drug. |
| Tan et al., (2011) | agonist | AM251 | amygdala | cross-sectional | inhibitory avoidance | pre-training administration↓ memory at 500 ng. Post-training does not impair memory at 500 ng |
| Wise et al., (2009) | agonist | CP55940 | hippocampus | cross-sectional | radial arm maze | ↓ memory performance. Effects blocked by rimonabant |
| Wegener et al., (2008) | agonist | WIN55,212-2 | intraperitoneal & nucleus accumbens shell, VTA, mPFC, hippocampus | cross-over | Eight arm radial arm maze | Peripheral dose of 1.2 WIN & central dose (5 ug/0.3 uL) into dorsal hippocampus ↓ memory |
| Yim et al., (2008) | agonist | WIN55,212-2 | intraperitoneal | cross-sectional | Morris water maze | agonist ↓ memory if drug given 4 weeks before test but no effect if drug given 1 week before test |
| Zarrindast et al., (2011) | agonist | WIN 55,212-2 | hippocampus | cross-sectional | inhibitory avoidance | ↓ performance at dose 0.5 ug/rodent |
| Zarrindast et al., (2012) | agonist | WIN 55,212-2 | hippocampus | cross sectional | inhibitory avoidance | ↓ performance at doses 0.1 and 0.25 |

ACPA= N-(Cyclopropyl)-5Z,8Z,11Z,14Z-eicosatetraenamide; THC=delta-9-tetrahydrocannabinol, CBD=cannabidiol, AM251= N-(Piperidin-1-yl)-5-(4-iodophenyl)-1-(2,4-dichlorophenyl)-4-methyl-1H-pyrazole-3-carboxamide; CP55,940= (-)-cis-3-[2-Hydroxy-4-(1,1-dimethylheptyl)phenyl]-trans-4-(3-hydroxypropyl)cyclohexanol; HU-210= (6aR)-trans-3-(1,1-Dimethylheptyl)-6a,7,10,10a-tetrahydro-1-hydroxy-6,6-dimethyl-6H-dibenzo[b,d]pyran-9-methanol; anandamide= N-(2-Hydroxyethyl)-5Z,8Z,11Z,14Z-eicosatetraenamide; SR141716A= N-(Piperidin-1-yl)-5-(4-chlorophenyl)-1-(2,4-dichlorophenyl)-4-methyl-1H-pyrazole-3-carboxamide hydrochloride; (R)-(+)-[2,3-Dihydro-5-methyl-3-(4-morpholinylmethyl)pyrrolo[1,2,3-de]-1,4-benzoxazin-6-yl]-1-naphthalenylmethanone mesylate; kg=kilogram; ug=microgram;

**Supplementary table 7. Non-human primate and monkey studies: sample characteristics**

| **Source** | **Journal** | **Age** | **Sex** | **Weight or BMI** | **N** | **Species** | **Cannabis use (lifetime/current)** |
| --- | --- | --- | --- | --- | --- | --- | --- |
| Aigner et al., (1988) | Psychopharmacology | Not reported | male | Mean not reported. Weight range=4.5-6.00 kg | 3 per group | Rhesus monkeys | N/A |
| Grilly et al., (1973) | Bulletin of the Psychonomic Society | Mean not reported. Adult=5, juvenile=8. | not reported | not reported | 13 | Chimpanzees | N/A |
| Taffe et al., (2012) | Journal of Psychopharmacology | Mean not reported. Age range=5-6 years | male | Mean not reported. Range=8-13.4 kg | 8 | Rhesus monkeys | N/A |
| Verrico et al., (2012) | Neuropsychopharmacology | M=23.9 months (adolescent) | male | not reported | 14 | Rhesus monkeys | N/A |
| Wright et al., (2013) | British Journal of Pharmacology | Mean not reported. Age range=12-13 years (adults) | male | Mean=14.7 kg | 10 | Rhesus monkeys | N/A |
| John et al., (2018) | Journal of Pharmacology and Experimental Therapeutics | Mean not reported. (adults) | male | not reported | 6 | Rhesus monkeys | N/A |
| Kangas et al., (2016) | Journal of Pharmacology and Experimental Therapeutics | Not reported | male | not reported | 9 | Squirrel Monkeys | N/A |
| Saletti et al., (2017) | Frontiers in Pharmacology | Not reported | male and female | Mean not reported. Range = 2.5-5.0 kg | 5 | Capuchin Monkeys | N/A |

N/A=not applicable

**Supplementary table 8. Non-human primate and monkey studies: sample characteristics: drug administration methods**

| **Source** | **Type of drug** | **Name of drug** | **Drug admin method** | **Dose** | **Type of administration: acute/chronic** | **When drug given relative to training** | **when drug given relative to test** |
| --- | --- | --- | --- | --- | --- | --- | --- |
| Aigner et al., (1988) | agonist | THC | oral | 4, 8, 16 mg/kg (1 and 2 hours prior to testing) | acute (2 days) & chronic (21 days) | post-training | 2 hours before test |
| Grilly et al., (1973) | agonist | THC vs placebo | oral | 1 mg/kg | acute | post-training | 2.5 hours before test |
| Taffe et al., (2012) | agonist | THC vs placebo | intramuscular | 0.1, 0.2 and 0.3 mg/kg | acute/sub-chronic (drug given 3 times at different doses) | post-training | 45 mins before test |
| Verrico et al., (2012) | agonist | THC vs placebo | intravenous | 30, 60, 120, 180, 240 ug/kg | sub-chronic (N=7 drug given 4 time; N=5 drug given 5 times) | post-training | 30 mins before test |
| Wright et al., (2013) | agonist and antagonist | THC vs vehicle, CBD ve vehicle, THC+CBD vs vehicle | intramuscular | 0.2, 0.5 mg/kg THC and 0.5 mg/kg CBD | acute | pre-training | 30 mins before test |
| John et al., (2018) | agonist | THC vs placebo | intravenous | 1.0 mg/kg for first 10 weeks, 2 mg/kg for last 2 weeks. | chronic (12 weeks) | post-training | 22 hours before test |
| Kangas et al., (2016) | agonist and antagonist | THC vs Rimonabant+THC, Anandamide | intravenous | 0.3ml/kg | acute | post-training | 5 minutes for anandamide, 30 minutes for THC |
| Saletti et al., (2017) | antagonist | CBD | intravenous | 15, 30, 60 mg/kg | acute | post-training | 30 mins before test |

THC=delta-9-tetrahydrocannabinol, CBD=cannabidiol; mins=minutes; mg=milligrams; kg=kilograms

**Table 9. Non-human primate and monkey studies: sample characteristics: key findings**

| **Source** | **Type of drug** | **Name of drug** | **Drug administration method** | **Design** | **Blinding** | **Memory paradigm** | **Results** |
| --- | --- | --- | --- | --- | --- | --- | --- |
| Aigner et al., (1988) | agonist | THC | oral | cross-over | double-blind | DMTS | ↓ Visual recognition memory performance at 2 and 4 mg/kg following acute & chronic (21 days) |
| Grilly et al., (1973) | agonist | THC vs placebo | oral | cross-over | single-blind | DMTS | ↓ memory performance accuracy but no effect on choice speed at 1 mg/kg |
| Taffe et al., (2012) | agonist | THC vs placebo | intramuscular | cross-over (3-4 days apart) | single-blind | Visuo-spatial paired associates learning test & CANTAB | ↓ memory performance at 0.2 and 0.3 mg/kg on the visuo-spatial paired associates learning test |
| Verrico et al., (2012) | agonist | THC vs placebo | intravenous | cross-over | single-blind | DMTS | ↓ memory performance on spatial memory task at 240 ug/kg following long delay (16s) but not short delay (6s) |
| Wright et al., (2013) | agonist and antagonist | THC vs vehicle, CBD ve vehicle, THC+CBD vs vehicle | intramuscular | cross-over (3-4 days apart) | open label | Paired associates learning test | THC ↓ memory performance at 0.2 and 0.5 mg. CBD (0.5 mg) blocked the effects of THC (0.2 or 0.5 mg) on memory |
| John et al., (2018) | agonist | THC vs placebo | intravenous | cross-over | single-blind | CANTAB | acute and chronic THC ↓ memory performance on DMS |
| Kangas et al., (2016) | agonist and antagonist | THC vs Rimonabant+THC, Anandamide | intravenous | cross-over | not reported | DMTS, | 0.03mg/kg THC No changes, at 0.01mg/kg produced ↓ in accuracy under the smaller delay values (0, 2, and 4 seconds)but not longer delays. 1.0mg/kg Rimonabant + 0.32mg/kg THC no effect, Anandamide No effect |
| Saletti et al., (2017) | antagonist | CBD | intravenous | cross-over (2 weeks apart) | not reported | PPI | CBD had no effect on PPI response |

THC=delta-9-tetrahydrocannabinol, CBD=cannabidiol; DMTS=Delayed match to sample task; CANTAB=Cambridge Neuropsychological test automated battery; PPI=pre-pulse inhibition task; mg=milligrams; kg=kilograms

**Table 10. Human studies: sample characteristics**

| **Source** | **Journal** | **Age** | **Sex** | **Weight** | **N** | **Subjects** | **Cannabis use** |
| --- | --- | --- | --- | --- | --- | --- | --- |
| Rabinak et al., (2014) | Neurobiology of Learning and Memory | Mean not reported. Age range=21-45 years | male and female | not reported | 14 per group | Humans | Lifetime use: less than 10 uses |
| Miller et al., (1977) | Pharmacology Biochemistry & Behaviour | Mean not reported. Age range=21-28 years | male | not reported | 34 | Humans | Current use: 2-4 times per week |
| Horder et al., (2009) | Psychopharmacology | M=24.1 years | male and female | not reported | 15 per group | Humans | Current use: not in last 7 days |
| Horder et al., (2009) | Psychopharmacology | M=24.1 years | male and female | not reported | 15 per group | Humans | Current use: not in last 7 days |
| Englund et al., (2013) | Journal of Psychopharmacology 27 (1), 19-27. | M=26 years | male and female | BMI=25 | vehicle=26, CBD=22 | Humans | Lifetime: more than 1 use, Current Use: Non in last 7 days |
| Ballard et al., (2013) | Psychopharmacology (Berl) | Mean not reported. Age range=18-35 years | male and female | not reported | 25 | Humans | Lifetime use: greater than 10 uses. Current use: mean 2.66 uses per month |
| Bossong et al., (2012) | Biological Psychiatry | M=21.4 years | male | M=75.6 kg | 17 | Humans | Lifetime use: at least 4 uses. M=18.1 uses per year; Current use: no use in last 2 weeks |
| Bossong et al., (2012) | Journal of Cognitive Neuroscience | M=21.6 years | male | M=78.7 kg | 13 | Humans | Lifetime use: greater than 4 uses; M= 17.0 uses per year; Current use: less than weekly use one year prior to inclusion |
| Darley et al., (1973) | Memory & Cognition | Mean not reported. All subjects adults. | male | not reported | 42 | Humans | Current use: not more than once per week |
| Dittrich et al., (1973) | Psychopharmacologia | Mean not reported. Age range=21-38 years | male and female | not reported | 37 | Humans | Lifetime use: none (n=15), between 4-25 uses (N=15) |
| Doss et al., (2018) | Biological Psychiatry | M=22.74 years | male and female | BMI = 24.62 | 23 | Humans | Lifetime use: 27.26 uses; current use: 0.78 uses per month |
| D'Souza et al., (2004) | Neuropsychopharmacology | M=29 years | male and female | 174.7 pounds | 22 | Humans | Lifetime use: no hx of cannabis use disorder. Current use: none in last week. |
| Hindocha et al., (2017) | Psychological medicine | M=24.46 years | male and female | not reported | 24 | Humans | Lifetime use: no hx of cannabis dependence; Current use: more than once per month but less than 3 times a week |
| Hooker et al., (1987) | Psychopharmacology | M=22.0 years | males | not reported | 12 | Humans | Lifetime use: less than twice a week |
| Keles et al., (2017) | Frontiers in Human Neuroscience | M=24.0 years | males and females | M=72.6 kg | 18 (13 analysed) | Humans | Current use: at least weekly cannabis use |
| Ranganathan et al., (2017) | Progress in Neuropsychopharmacology & Biological Psychiatry | M=25.7 years | males and females | not reported | 38/57 | Humans | Lifetime use: included hx cannabis dependence |
| Ranganathan et al., (2014) | Psychopharmacology | M=23.1 years | male and female | M=162 pounds | 30 | Humans | Lifetime use: more than one use but less than 50 uses ( ? Lifetime M= 54.8 use) |
| Solowij et al., (2018) | Cannabis and Cannabinoid Research | M=25.1 years | male and female | BMI = 22.68 | 20 | Humans | Current use: yes - frequent cannabis users ( last month M= 25 days) |
| Theunissen et al., (2018) | British Journal of Pharmacology | M=23.5 years | male and female | M=66.67 kg | 6 | Humans | Current use: yes |
| Das et al., (2013) | Psychopharmacology | Mean not reported. Age range=18-35 years | male and female | not reported | 48 | Humans | Current use: yes - 1-3 uses per month |
| Horder et al., (2012) | Journal of Psychopharmacology | M=21.6 years | male and female | BMI=22.7 | 21 | Humans | Current use: not in last 7 days |
| Englund et al., (2016) | Journal of Psychopharmacology | M=23.8 years | male | BMI=22.69 | 10 | Humans | Lifetime use: less than 25 uses |
| Bhaattacharyya et al., (2009) | Archives of General Psychiatry | M=26.7 years | male | not reported | 15 | Humans | Lifetime use: less than 15 uses Current Use: None in last month |
| Bhaattacharyya et al., (2010) | Neuropsychopharmacology | M=26.7 years | male | not reported | 15 | Humans | Lifetime use: less than 14 times |
| Curran et al., (2002) | Psychopharmacology | M=24.2 years | male | not reported | 15 | Humans | Lifetime use: more 1 but no current use more than once per week |
| D'Souza et al., (2008) | Psychopharmacology (Berl) | M=24.89 years | not reported | M =159.35 | 28 | Humans | group 1 (N=17): no recent history of substance abuse in last 3 months; group 2 (N=11): Lifetime use: greater than 100 times; Current use: use within last week, exposure greater than 10 times per month |
| D'Souza et al., (2005) | Biological Psychiatry | M=29.00 years | male and female | M=174.7 | 22 | Humans | Lifetime use: at least once but less than 3 months of abuse/dependence |
| D'Souza et al., (2008) | Neuropsychopharmacology | Group 1 M=29 years; Group 2 M=24.8 years | male and female | Group 1 M=174.7, Group 2 M =165.7 | 52 | Humans | group 1 (N=22) non-users (published in 2005); group 2 (N=30): frequent cannabis users |
| Heishman et al., (1990) | Pharmacology, Biochemistry and Behaviour | Mean not reported. Age range=27-29 years | male | not reported | 3 | Humans | Current use: 4.7 joints per month |
| Heishman et al., (1989) | Pharmacology, Biochemistry and Behaviour | M=31.1 years | male | not reported | 12 | Humans | Current use: yes - 83% of sample |
| Hunault et al., (2014) | Psychopharmacology | M=24.1 years | male | BMI=22.1 | 24 | Humans | Current use: yes - 2-9 joints per month |
| Leweke et al., (1998) | Neuropsychobiology | M=27.2 years | male | not reported | 19 | Humans | Lifetime use: yes; Current use: no |
| Mathai et al., (2018) | Journal of Neuropsychiatry and Clinical Neuroscience | M=35.0 years | not reported | not reported | 10 | Humans | Lifetime use: yes; Current use 27.8 uses per day in last month |
| Miller et al., (1978) | Pharmacology, Biochemistry and Behaviour | Mean not reported. Age range=21-28 years. | male | not reported | 16 | Humans | Current use: 2-4 times per week |
| Makela et al., (2006) | Neuropsychopharmacology | M=21.8 years | male and female | not reported | 19 | Humans | Lifetime: yes {<10 = 6 people, 10-100 = 10 people, >100 = 3} |

**THC=delta-9-tetrahydrocannabinol, CBD=cannabidiol; M=mean; SD=standard deviation**

**Table 11. Human studies: drug administration methods**

| **Source** | **Type of drug** | **Name of drug** | **Drug administration method** | **Dose** | **Type of administration: acute/chronic** | **When drug given relative to training** | **when drug given relative to test** |
| --- | --- | --- | --- | --- | --- | --- | --- |
| Rabinak et al., (2014) | agonist | THC vs placebo | oral | 7.5 mg/kg | acute | post-training | 120 mins before test |
| Miller et al., (1977) | agonist | THC vs placebo | inhalation | 1.4% (14mg) | acute | pre-training | immediately after smoking |
| Horder et al., (2009) | antagonist | Rimonabant | oral | 20 mg | acute | pre-training | 2.5 hours before test |
| Horder et al., (2009) | antagonist | Rimonabant | oral | 20 mg | acute | pre-training | 2.5 hours before test |
| Englund et al., (2013) | agonist and antagonist | CBD vs placebo, CBD+THC vs placebo | oral CBD, intravenous THC | 600 mg CBD, 1.5mg THC | acute | pre-training | CBD vs. placebo: 2.5 hours before test. THC vs. placebo: 1 hour 10 mins before test |
| Ballard et al., (2013) | agonist | THC vs placebo | Oral | 7.5, 15 mg | sub-chronic | pre-training | 90 mins before test |
| Bossong et al., (2012) | agonist | THC vs placebo | inhalation | 6 mg (dose 1), 1 mg (dose 2), 1 mg (dose 3) - all on same day | acute | pre-training | not reported |
| Bossong et al., (2012) | agonist | THC vs placebo | inhalation | 6 mg (dose 1), 1 mg (dose 2), 1 mg (dose 3) - all on same day | acute | pre-training | not reported |
| Darley et al., (1973) | agonist | THC vs placebo | oral | 20 mg | acute | post-training | 1 hour before test |
| Dittrich et al., (1973) | agonist | THC vs placebo | oral | 15 mg | acute | pre-training | 2 hours before test |
| Doss et al., (2018) | agonist | THC vs placebo | oral | 15 mg | acute | post-training | 48 hours post-training, 120 min before test |
| D'Souza et al., (2004) | agonist | THC vs placebo | intravenous | 2.5mg, 5mg | sub-chronic (3 days) | pre-training | 30min before test |
| Hindocha et al., (2017) | agonist | Bedrobinol (16% THC and less than 1% CBD) vs placebo (0.07% THC) | inhalation | 66.67 mg | acute | post-training | 35 mins before 1st IR test; 55 mins before N-Back |
| Hooker et al., (1987) | agonist | THC vs Placebo | inhalation | 10 mg | acute | post-training | testing prior to thc & 15 mins after thc |
| Keles et al., (2017) | agonist | Dronabinol vs. placebo | oral | 50 mg | acute | post-training | 1.5 (~2hr) hours before test |
| Ranganathan et al., (2017) | agonist | THC vs placebo | intravenous | 0.03 mg/kg ( Experiement 2 0.05mg/kg) | acute | post-training (#1 pre-encoding) | 35 mins before and 25 mins after THC |
| Ranganathan et al., (2014) | agonist | THC vs placebo | intravenous | 0.018, 0.036 mg/kg | acute | post-training | 40 and 70 mins before test |
| Solowij et al., (2018) | antagonist | CBD | oral | 200 mg daily for 10 weeks | chronic (10 weeks) | post-training | not reported |
| Theunissen et al., (2018) | agonist | JWH-018 vs placebo | inhation | 2 and 3 mg | acute (drug given twice) | post-training | 30 mins before test |
| Das et al., (2013) | antagonist | CBD vs placebo before extinction, CBD vs placebo prior to extinction | inhalation | 32 mg | acute | pre-extinction group: fear conditioning, drug given, fear extinction task. Post-extinction group: fear conditioning, fear extinction, drug. Prose test pre-training | 5 mins post drug administration (fear extinction), 24 hours post drug administration (delayed prose recall) |
| Horder et al., (2012) | antagonist | Rimonabant vs. placebo | oral | 20 mg | sub-chronic (7 days) | pre-training | 2.5 hours before test |
| Englund et al., (2016) | agonist | THCV vs placebo | oral THCV | 10 mg THCV (5 days) and 9 ml THC (1 day) | sub-chronic (4 days THC and 1 day THC) | pre-training | 10-25 minutes before test |
| Bhaattacharyya et al., (2009) | agonist & antagonist | THC, CBD vs. placebo | oral | 10 mg THC, 600 mg CBD | acute | post-training | 1 hour before test |
| Bhaattacharyya et al., (2010) | agonist & antagonist | THC, CBD vs. placebo | oral | 10 mg THC, 600 mg CBD | acute | post-training | 1 hour before test |
| Curran et al., (2002) | agonist | Dronabinol vs placebo | oral | 7.5 and 15 mg | acute | post-training | 1 hour before test |
| D'Souza et al., (2008) | agonist | THC vs placebo | intravenous | 0.0286 mg/kg | acute | post-training | 30 mins before test |
| D'Souza et al., (2005) | agonist | THC vs placebo | intravenous | 2.5, 5 mg | acute | post-training | 30 mins before test |
| D'Souza et al., (2008) | agonist | THC vs placebo | intravenous | 2.5, 5 mg | acute | post-training | 30 mins before test |
| Heishman et al., (1990) | agonist | THC vs placebo | inhalation | 1 or 2 cigarettes containing 2.57% THC | acute | pre-training | 10 mins before test |
| Heishman et al., (1989) | agonist | THC vs placebo | inhalation | 13 or 27% THC cigarettes 12 /21mg THC | acute | pre-training | 5 mins before test |
| Hunault et al., (2014) | agonist | THC vs placebo | inhalation | 29.3, 49.1, 69.4 mg | sub-chronic (4 days) | post-training | 35 mins before test |
| Leweke et al., (1998) | agonist | Dronabinol vs placebo | inhalation | 10 mg | acute | pre-training | 150 minutes before test |
| Mathai et al., (2018) | agonist | THC vs placebo | oral | 10, 20 mg | chronic (7 days) | post-training | 1 hour after 20 mg dose |
| Miller et al., (1978) | agonist | THC vs placebo | inhalation | 5, 10, 15 mg | acute | pre-training | immediately after smoking |
| Makela et al., (2006) | agonist | THC vs placebo | Sublingual Spray | 5mg | acute | pre-training | 120 mins before test |

THC=delta-9-tetrahydrocannabinol, CBD=cannabidiol; thcv=tetrahydrocannabivarin; mins=minutes; mg=milligrams

**Table 12. Human studies: key findings**

| **Source** | **Type of drug** | **Name of drug** | **Design** | **Blinding** | **Memory paradigm** | **Results** |
| --- | --- | --- | --- | --- | --- | --- |
| Rabinak et al., (2014) | agonist | THC vs placebo | cross-sectional | double-blind | memory extinction | no effect |
| Miller et al., (1977) | agonist | THC vs placebo | cross-sectional | not blinded | Immediate Free Recall; Final Free Recall; Delayed recognition memory test | ↓ short-term memory performance at 14 mg/kg |
| Horder et al., (2009) | antagonist | Rimonabant | cross-sectional | double-blind | Rey Auditory Verbal Learning Task | No difference between drug and placebo |
| Horder et al., (2009) | antagonist | Rimonabant | cross-sectional | double-blind | Word catergorisation and memory | ↓ word recall for self referent positive words at 20 mg |
| Englund et al., (2013) | agonist and negative allosteric modulator | CBD vs placebo, CBD+THC vs placebo | cross-over (same day) | double-blind | Hopkins Verbal Learning Task-Revised; Digit span forward and reverse | THC ↓ memory performance on immediate and delayed recall. No effect on immediate recall following THC+CBD vs placebo. THC+CBD vs placebo ↑ memory performance on delayed recall. |
| Ballard et al., (2013) | agonist | THC vs placebo | cross-over | double-blind | Emotional memory task | ↓ memory performance for emotive pictures at 7.5 and 15 mg |
| Bossong et al., (2012) | agonist | THC vs placebo | cross-over (2 weeks apart) | double-blind | Sternberg working memory paradigm | ↓ memory performance at 8 mg at working memory load 3 and 5 |
| Bossong et al., (2012) | agonist | THC vs placebo | cross-over | double-blind | Associative memory | no effect on performance. Drug caused reduced functional activation in neural correlates of memory encoding |
| Darley et al., (1973) | agonist | THC vs placebo | cross-sectional | double-blind | Immediate free word recall, Delayed recall 1 hour post-testing | ↓ memory performance at 20 mg |
| Dittrich et al., (1973) | agonist | THC vs placebo | cross-over | double-blind | Baddley and Warrington (1970) memory test | ↓ immediate recall retention for words at front/middle of list at 15 mg, No effect of drug vs. placebo for last words in the list. ↓ input into longterm memory |
| Doss et al., (2018) | agonist | THC vs placebo | cross-over | double-blind | Emotional memory task & False memory task | ↓ memory performance at 15 mg on False memory test. No effect on emotional memory test |
| D'Souza et al., (2004) | agonist | THC vs placebo | cross-over (1 week apart) | double-blind | Hopkins Verbal Learning Test | ↓ memory performance (immediate & delayed recall). No effect on recognition recall. WM ↓ accuracy performance for easy subtask |
| Hindocha et al., (2017) | agonist | Bedrobinol (16% THC and less than 1% CBD) vs placebo (0.07% THC) | cross-over | double-blind | Spatial N-back working memory test, Prose recall rivermead behavioural memory test | ↓ memory performance at 66.67 mg on 1 and 2 back of N-back test. ↓ memory performance at 66.67 mg on prose delayed recall but not prose immediate recall |
| Hooker et al., (1987) | agonist | THC vs Placebo | cross-over (1-3 weeks apart) | double-blind | Randt Memory Battery | No effect on immediate or delayed free recall for semantic memory. ↓ memory performance on recall of word lists/short story (greater omissions & intrusions) |
| Keles et al., (2017) | agonist | Dronabinol vs. placebo | cross over (same day) | double-blind | N-back working memory test | No effect on memory performance. fMRI BOLD signal greater following THC vs. placebo in 2-back of working memory test. |
| Ranganathan et al., (2017) | agonist | THC vs placebo | cross-over (2-3 weeks apart) | double-blind | Rey Auditory Verbal Learning Task | ↓ memory performance at 0.05 mg/kg (#2) on immediate , short and long-delayed recall, No significant difference for post -encoding THC (#1) |
| Ranganathan et al., (2014) | agonist | THC vs placebo | cross-over | double-blind | Rey Auditory Verbal Learning Task | ↓ memory performance on immediate and long-delayed recall in dose dependent manner . ↑ total errors for spatial working memory |
| Solowij et al., (2018) | Negative allosteric modulator | CBD | cross-over | open label | Rey Auditory Verbal Learning Task | ↑ memory performance at 200 mg on words recalled (trials 1-5) & recall postinteference. No effect on delayed recall. |
| Theunissen et al., (2018) | agonist | JWH-018 vs placebo | cross-over | single-blind | Spatial memory task | No effect at 2 or 3 mg |
| Das et al., (2013) | Negative allosteric modulator | CBD vs placebo before extinction, CBD vs placebo prior to extinction | cross-over | double-blind | Fear conditioning task, fear extinction task, fear recall task | Extinction: No effect of CBD pre-extinction vs. vehicle. CBD post-extinction reduced expectancy ratings vs placebo. |
| Horder et al., (2012) | antagonist | Rimonabant vs. placebo | cross-over | double-blind | Rey Auditory Verbal Learning Task and Word categorisation test. | No effect on memory performance on Rey AVLT. Rimonabant vs placbeo reduced number of intrusion errors (novel words falsely endorsed as previously seen) for self-relevant and non-self relevant words. |
| Englund et al., (2016) | agonist | THCV or THC vs placebo | cross-over | double-blind | Hopkins Verbal learning test, immediate/delayed recall | No effect of THCV on Hopkins verbal learning test, immediate/delayed recall |
| Bhaattacharyya et al., (2009) | agonist & negative allosteric modulator | THC, CBD vs. placebo | cross-over | double-blind | Verbal Paired associates task | No effect of either drug on verbal learning performance. |
| Bhaattacharyya et al., (2010) | agonist & negative allosteric modulator | THC, CBD vs. placebo | cross-over | double-blind | Verbal memory task | same data as 2009 |
| Curran et al., (2002) | agonist | Dronabinol vs placebo | cross-over (1 week apart) | double-blind | Prose recall (immediate and after 45 minutes) | ↓ memory performance at 15 mg on immediate/delayed recall. |
| D'Souza et al., (2008) | agonist | THC vs placebo | cross-over | double-blind | Hopkins Verbal Learning test, SWM, DMTS and CANTAB | ↓ memory performance on immediate, delayed, recall. ↑ between- errors for spatial working memory |
| D'Souza et al., (2005) | agonist | THC vs placebo | cross-over | double-blind | Hopkins Verbal Learning test | ↓ memory performance at 2.5 and 5 mg on immediate & delayed, recall. |
| D'Souza et al., (2008) | agonist | THC vs placebo | cross-over | double-blind | Hopkins Verbal Learning test | ↓ memory performance at 2.5 and 5 mg on immediate & delayed, recall in both groups. Frequent users showed poorer baseline performance and fewer THC induced deficits on immediate/delayed memory relative to non-users. |
| Heishman et al., (1990) | agonist | THC vs placebo | cross-over | double-blind | digit recall | ↓ memory performance on digit recall |
| Heishman et al., (1989) | agonist | THC vs placebo | cross-over | double-blind | WAIS digit span test | ↓ memory performance at 27% THC vs placebo |
| Hunault et al., (2014) | agonist | THC vs placebo | cross-over | double-blind | Visual analogue scale of self-reported memory impairment | ↓ memory performance at 29, 49 and 69 mg |
| Leweke et al., (1998) | agonist | Dronabinol vs placebo | cross-over (10 days apart) | double-blind | Word recognition test | ↓ memory accuracy performance at 10 mg |
| Mathai et al., (2018) | agonist | THC vs placebo | cross-over | single-blind | Hopkins Verbal Learning test | No effect on immediate/delayed learning |
| Miller et al., (1978) | agonist | THC vs placebo | cross-over (1 week apart) | double-blind | Immediate free recall, delayed recall, delayed recognition tests | ↓ memory performance on immediate and delayed recall |
| Makela et al., (2006) | agonist | THC vs placebo | cross-over (1 week apart) | double-blind | Spatial Working Memory Task | ↓ between-search errors in females at 8 boxes. |

THC=delta-9-tetrahydrocannabinol, CBD=cannabidiol; thcv=tetrahydrocannabivarin; mins=minutes; mg=milligrams

#### Memory paradigms used in rodent studies

Behavioural tasks used to investigate non-spatial memory in rodents typically included inhibitory avoidance; novel object detection and memory extinction tasks, which capture short-term memory formation and retrieval in vivo. Memory performance on the inhibitory avoidance paradigm is quantified as the response latency to approach a particular cue/context after the rodent had been conditioned to associate a particular cue/context with an aversive stimulus (i.e. electric foot shock). A decrease in this response latency is usually interpreted as reflecting impaired short-term memory performance. In contrast, the novel object detection task relies on quantifying the time a rodent spends exploring novel vs. familiar objects. Since rodents naturally show a preference for novelty, the time spent exploring familiar objects is thought to index poor memory performance. Pre-clinical paradigms used to investigate extinction resemble memory paradigms used in vivo to measure forgetting. Extinction paradigms typically involve 1) measuring the response latency to approach a cue/context after the rodent has been conditioned to associate an aversive stimulus with the cue and 2) measuring response latencies to approach a cue/context when the conditioned cue/context is presented in the absence of the aversive stimulus. Increased response latencies to approach aversive stimuli in this context are thought to index poor forgetting.

Pre-clinical paradigms used to investigate short-term spatial memory include the Morris water maze, eight arm radial maze and the Y maze. In the Morris water maze, rodents are required to find the spatial location of an escape platform, which is underwater inside an opaque water tank. The task may be varied involve the use of spatial cues outside of the tank or not. The time spent to find the escape platform (or time spent in the quadrant where it previously was – see probe trial) is used to index spatial memory learning and accurate retention/recall. In the radial arm maze, rewards are placed in certain arms of the maze and spatial working memory performance is quantified as the number of times the rodent enters those arms containing rewards, compared to those where no rewards are present. An increase in the number of entries into arms that do not contain rewards is thus thought to index poorer spatial memory performance. By contrast, the Y maze quantifies spatial working memory by examining exploration patterns made in a maze that does not contain rewards. Since rodents show a preference for novelty, the time spent exploring arms that are relatively more familiar are thought to index memory performance.

*Memory paradigms used in monkey and non-human primate studies*The delayed matching-to sample task is a working memory test that involved the presentation of a stimulus (i.e. illumination of one of three items on a press-plate manupulanda with one of seven geometric shapes). After the press-plate is pushed at random, the geometric figure disappears. Following an interval, three geometric shapes are presented. If the original geometric shape is correctly selected, a reward is administered; if an incorrect shape is selected, there is a 10 second timeout and a new trial begins.

#### Memory paradigms used in human studies

Behavioural tasks used to investigate short-term, verbal memory in humans primarily assessed the recall of verbal information. The memory test developed by Miller and colleagues (37) involves reading a short story to volunteers (200 words) and instructing volunteers to recall it immediately and after 24 hours. In contrast, the Hopkins Verbal Learning task involves reading volunteers a 12-item word list and instructing them to identify the words that were read from a 24-item word list which includes the aforementioned 12-item word list and a 12-item distractor word list (22). Similarly, the Rey Auditory Verbal Learning Task involves reading volunteers a 15-item word list, asking volunteers to immediately recall it, reading volunteers a second, 15-item, distractor word list and then asking volunteers to recall the first 15-item word list (38). A behavioural task used to investigate memory extinction in humans is the fear conditioning extinction paradigm (39). On day 1, volunteers are conditioned to associate a neutral visual stimulus with an aversive noise. On day 2, volunteers undergo extinction training where they are presented with the visual stimuli presented on day 1 in the absence of the aversive stimulus. On day 3, volunteers are presented with the visual stimuli including the stimuli presented on day 1 and asked to indicate which items might be linked to an aversive noise. Conditioned fear responses are indexed using skin conductance responses and expectancy ratings of whether the visual items will be linked with an aversive noise.


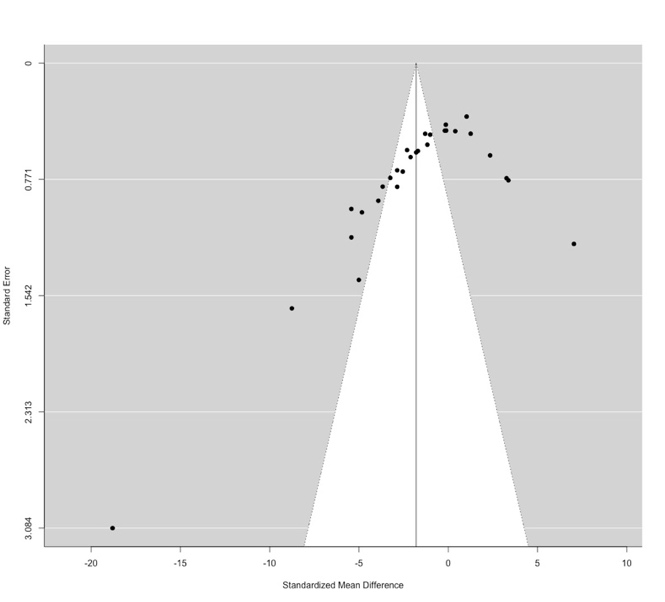


Supplementary figure 1. Funnel plot from meta-analysis of the acute effects of CB1R agonists relative to vehicle on non-spatial memory performance in rodents (g=-1.79, 95% confidence interval (CI), -3.13 to -0.45, p=0.009).


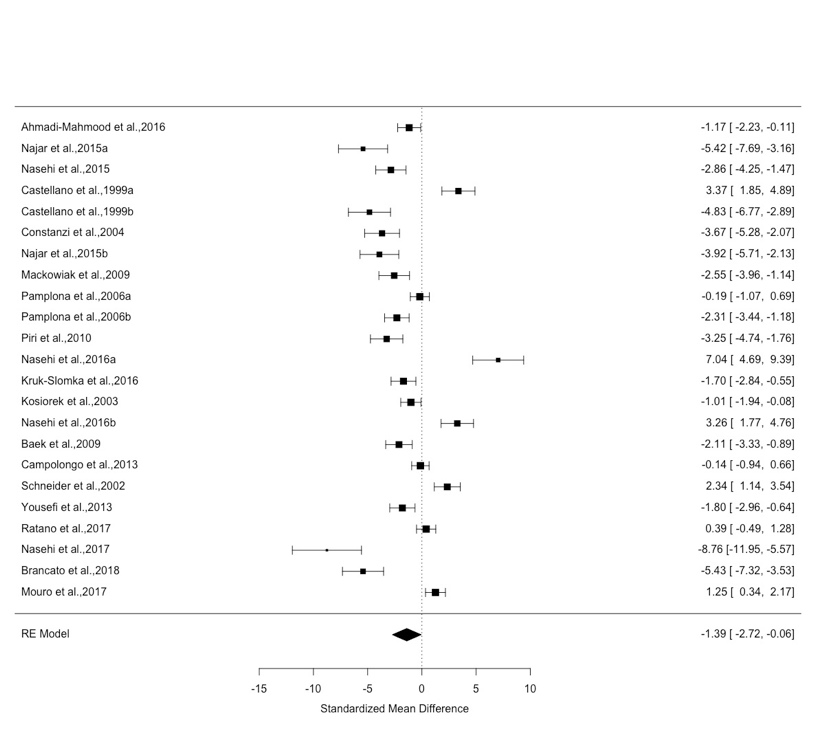

Supplementary figure 2. Forest plot from meta-analysis of the effects of full CB1R agonists relative to vehicle on non-spatial memory performance (g=-1.39, 95% confidence interval (CI), -2.72 to -0.06)


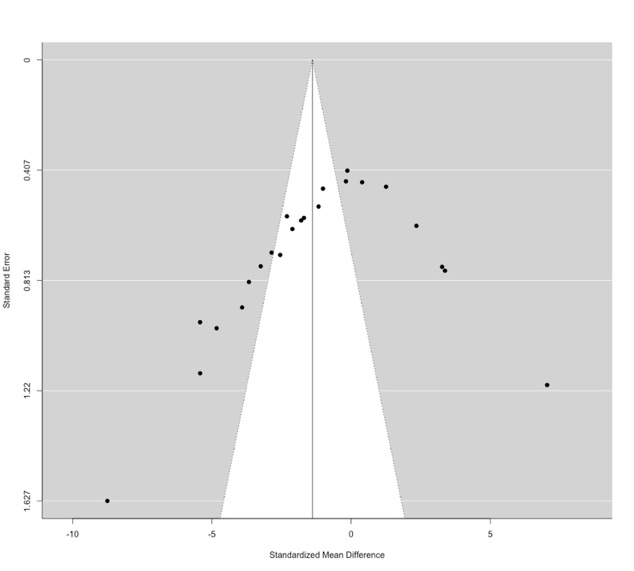


Supplementary figure 3. Funnel plot from meta-analysis of the effects of full CB1R agonists relative to vehicle on non-spatial memory performance (g=-1.39, 95% confidence interval (CI), -2.72 to -0.06)


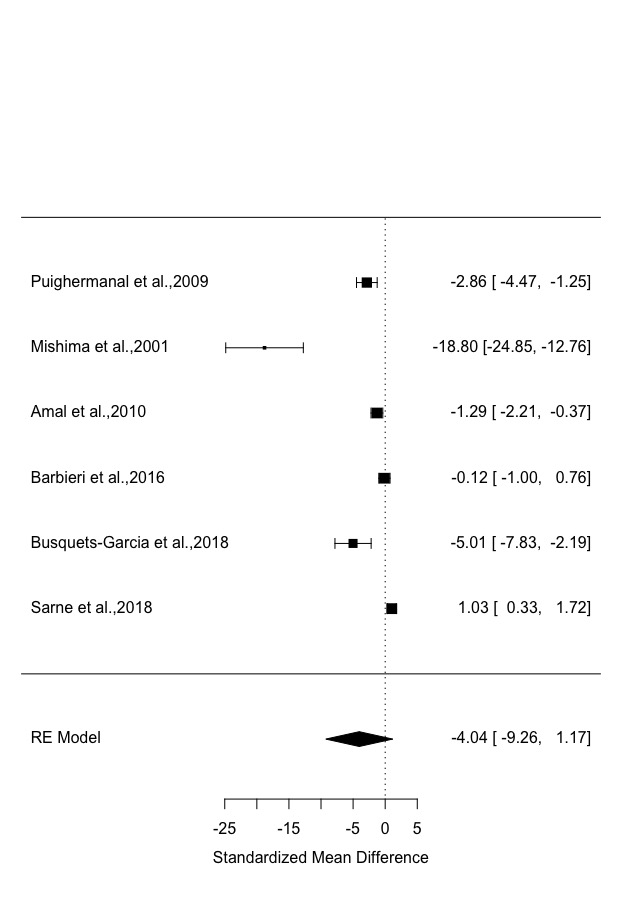


Supplementary figure 4. Forest plot from meta-analysis of the effects of partial CB1R agonists, delta-9-tetrahydrocannabinol relative to vehicle on non-spatial memory performance (g=4.04, 95% confidence interval (CI), -9.26 to 1.17, p=0.13).


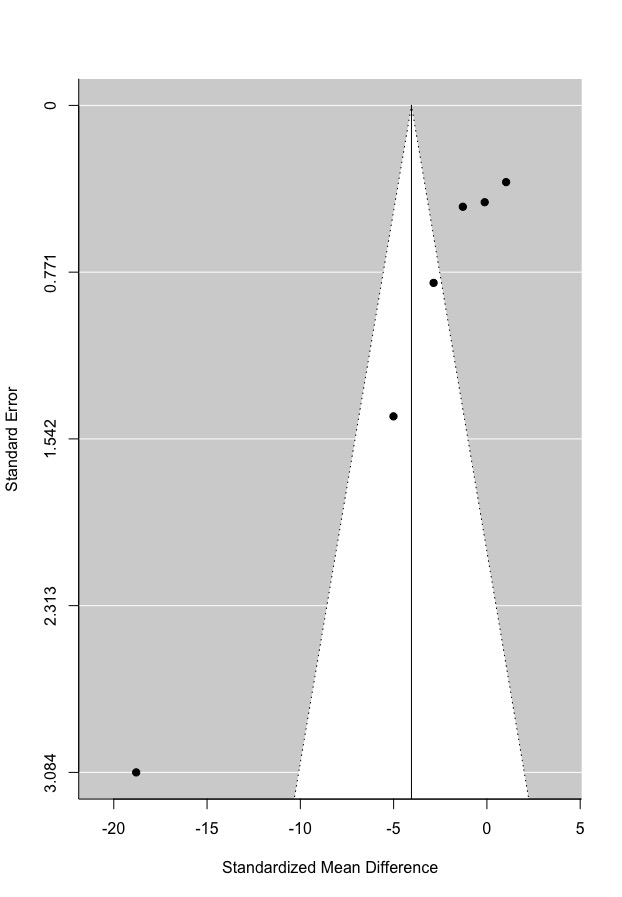


Supplementary figure 5. Funnel plot from meta-analysis of the effects of partial CB1R agonists, delta-9-tetrahydrocannabinol relative to vehicle on non-spatial memory performance (g=4.04, 95% confidence interval (CI), -9.26 to 1.17, p=0.13).


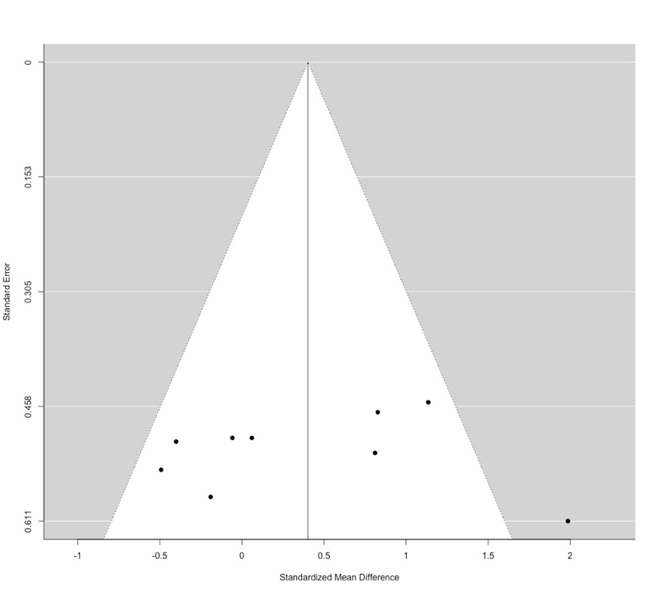


Supplementary figure 6. Funnel plot from meta-analysis of the effects of CB1R antagonists relative to vehicle on non-spatial memory performance in rodents (g=0.40, 95% confidence interval (CI), -0.11 to 0.92, p=0.12).


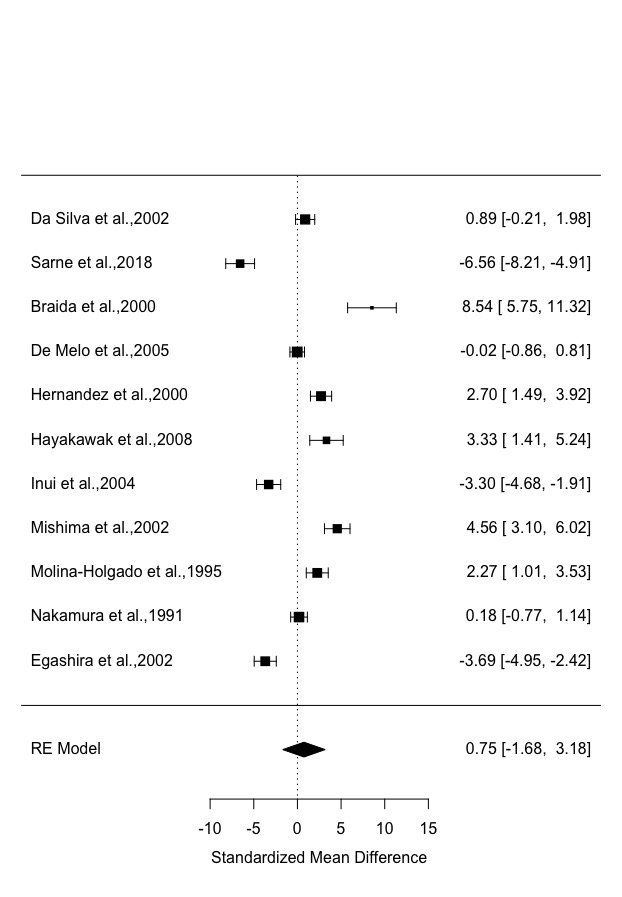


Supplementary figure 7. Forest plot from meta-analysis of the effects of CB1R agonists relative to vehicle on spatial memory performance in rodents (g=0.75, 95% confidence interval (CI), -1.68 to 3.18, p=0.55)


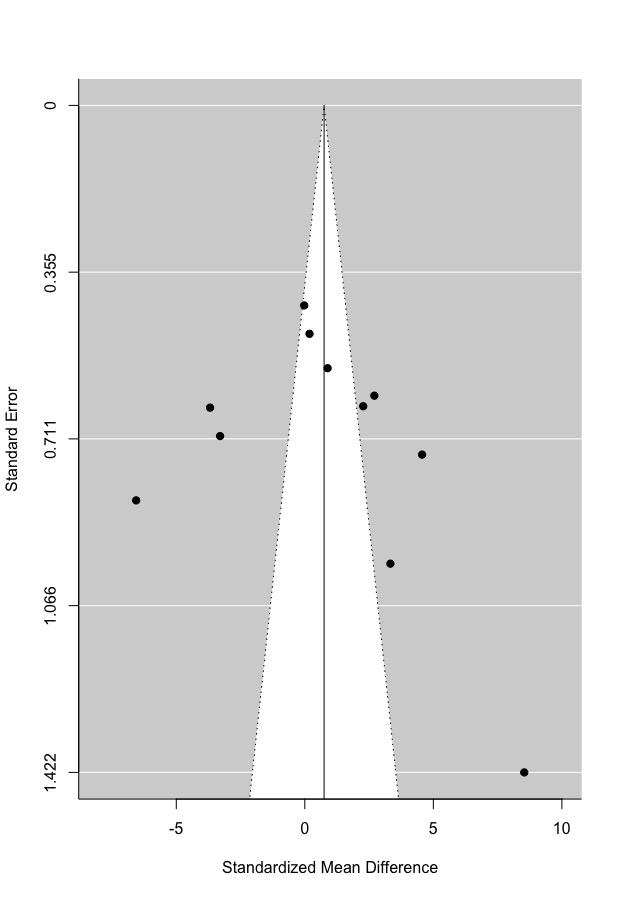


Supplementary figure 8. Funnel plot from meta-analysis of the effects of CB1R agonists relative to vehicle on spatial memory performance in rodents (g=0.75, 95% confidence interval (CI), -1.68 to 3.18, p=0.55)


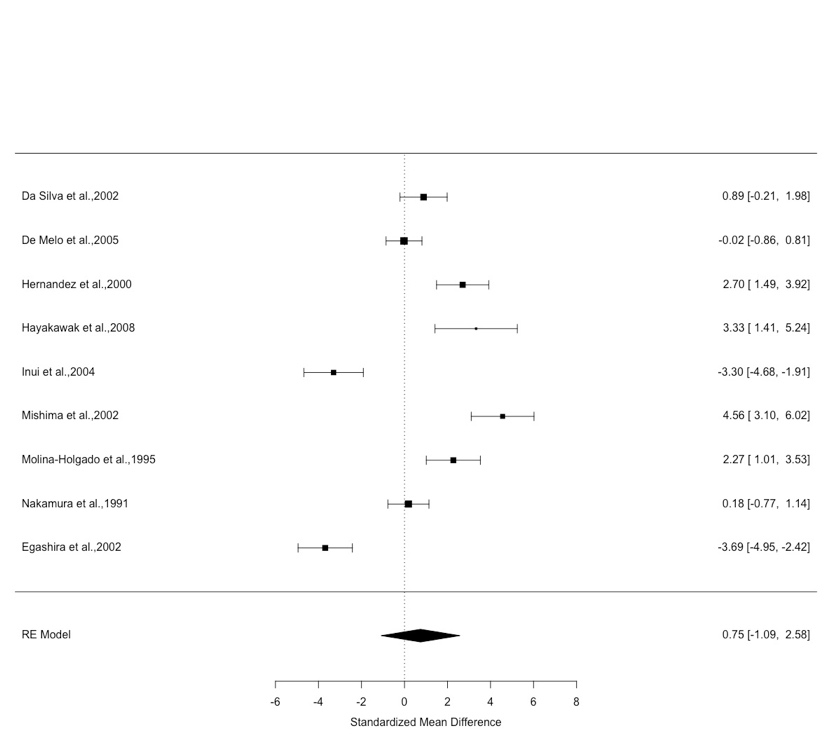


Supplementary figure 9. Forest plot from meta-analysis investigating the effects of partial CB1R agonist, THC, relative to vehicle (g=0.75, 95% confidence interval (CI), -1.09 to 2.58, p=0.43).


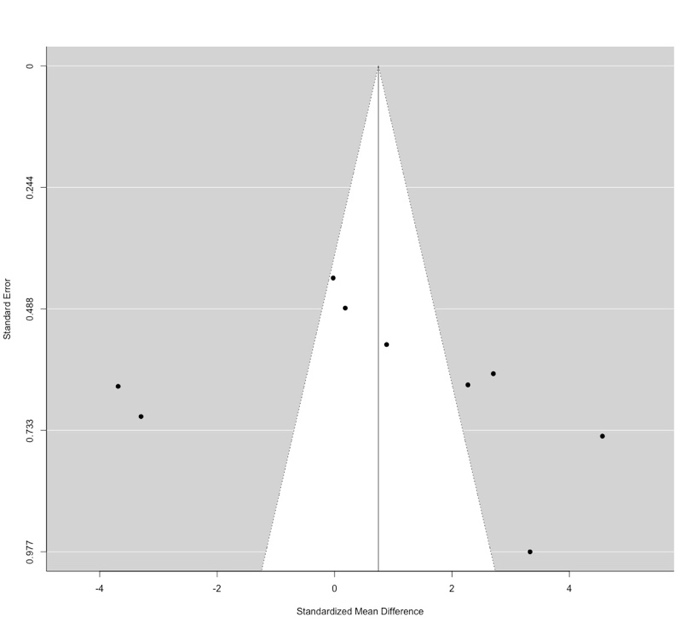


Supplementary figure 10. Funnel plot from meta-analysis investigating the effects of partial CB1R agonists relative to vehicle (g=0.75, 95% confidence interval (CI), -1.09 to 2.58, p=0.43).


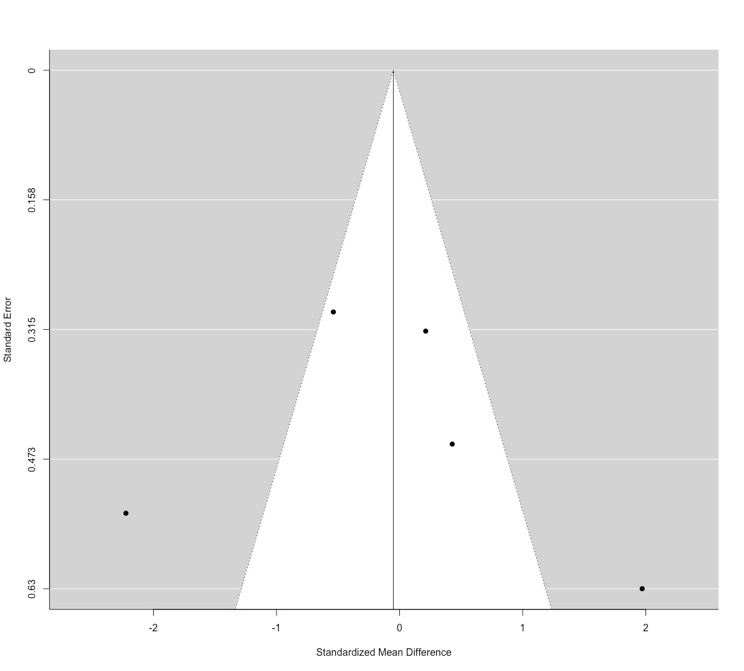


Supplementary figure 11. Funnel plot from meta-analysis of the chronic effects of CB1R agonists relative to vehicle on non-spatial memory performance in rodents (g=-0.05, 95% confidence interval (CI), -1.32 to 1.22, p=0.94)


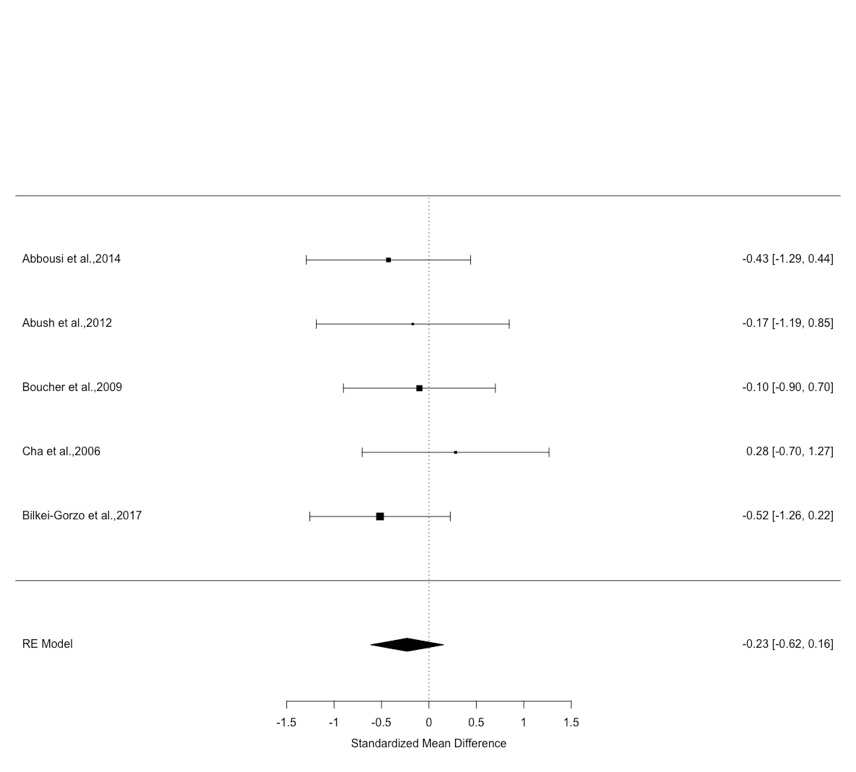


Supplementary figure 12. Forest plot from meta-analysis of the chronic effects of CB1R agonists relative to vehicle on spatial memory performance, as determined by the Morris water maze, in rodents (g=-0.23, 95% confidence interval (CI), -0.62 to 0.16, p=0.24


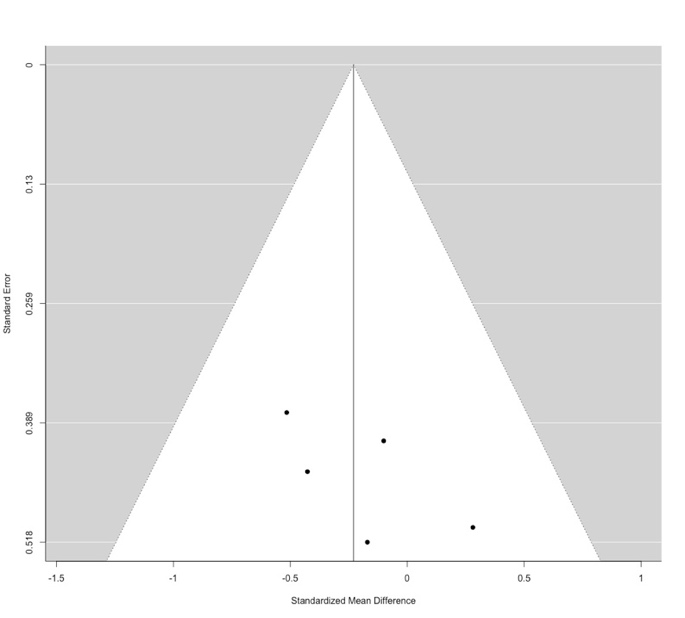


Supplementary figure 13. Funnel plot from meta-analysis of the chronic effects of CB1R agonists relative to vehicle on spatial memory performance, as determined by the Morris water maze, in rodents (g=-0.23, 95% confidence interval (CI), -0.62 to 0.16, p=0.24).


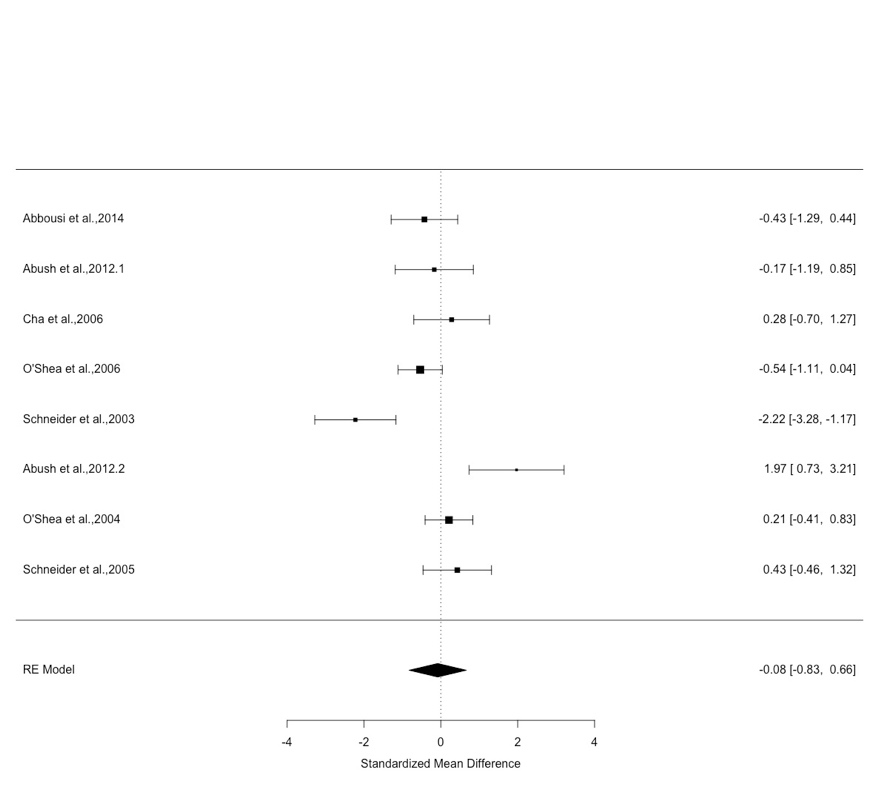


Supplementary figure 14. Forest plot from meta-analysis of the chronic effects of CB1R agonists relative to vehicle on spatial memory performance in rats (g=-0.08, 95% confidence interval (CI), -0.83 to 0.66, p=0.83).


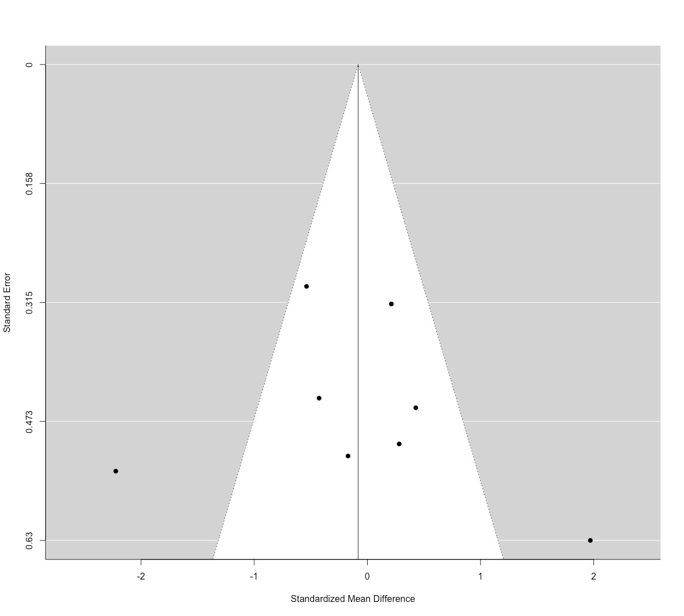


Supplementary figure 15. Funnel plot from meta-analysis of the chronic effects of CB1R agonists relative to vehicle on spatial memory performance in rats (g=-0.08, 95% confidence interval (CI), -0.83 to 0.66, p=0.83).
